# Application of metasurface in future displays

**DOI:** 10.1515/nanoph-2025-0269

**Published:** 2025-10-24

**Authors:** Lingyu Ai, Zhi Gan, Christoph Vannahme, Xiaolong Zhu

**Affiliations:** School of Integrated Circuits, 66374 Jiangnan University, Wuxi 214122, China; Department of Micro and Nanotechnology, Technical University of Denmark, DK-2800 Kongens Lyngby, Denmark; State Key Laboratory of Precision Spectroscopy, School of Physics and Electronic Science, East China Normal University, Shanghai 200241, China

**Keywords:** metasurface, metalens, 3D display

## Abstract

Optical metasurfaces, as a booming research field, have provided new methods for modulating the amplitude, phase, and polarization of light through artificial birefringent structures or structural resonances. It has been used to design planar optical components such as ultra-thin lenses, ultra-wideband achromatic lenses, and orbital angular momentum (OAM) generators. However, existing surveys typically examine either metasurface fundamentals or a single display modality, leaving no comprehensive roadmap that connects meta-atom design to full-device performance, hereafter, the term meta-atom are denoted to be an individual sub-wavelength building block of a metasurface. Here we present the first cross-scale review that quantitatively bridgices phase-dispersion engineering at the nanostructure level with system-level figures-of-merit across three mainstream 3D display paradigms, computer-generated holography, light-field projection, and near-eye/retinal displays. By critically benchmarking more than 150 demonstrations published between 2019 and 2025, we extract practical lookup charts that guide practitioners from material choice and meta-atom geometry to field-of-view, depth acuity, efficiency, and form-factor targets. Thanks to metasurfaces’ high integration density and functional diversity, its application in the light field display has attracted great interest. Metasurface can effectively improve the shortcomings of low spatial resolution, low diffraction efficiency, and narrow field of view common in traditional display components. In this paper, we first review the phase modulation method and structure resonance principle of metasurface. Then, we examine their application in the holographic display field and review the approaches for achieving structural-color printing. We summarize the 3D display methods of holographic display, light field display, and near-eye display and discuss how metasufaces enhance each modality. Finally, we distill emerging inflection points: AI assisted inverse design, dynamically tunable multifunctional platforms, and quantum or cascaded architectures into a looking forward commercialization roadmap that addresses the challenges still facing the 3D display industry.

## 1 Introduction

Metasurface, a planar branch of three-dimensional (3D) metamaterials [1], [2], [3], usually comprises subwavelength metal or dielectric material nanostructures. While retaining the extraordinary physical properties and electromagnetic functions of artificial materials, metasurface has the characteristics of reducing absorption loss and simplifying the manufacturing process. It can also manipulate the fundamental properties of light on the subwavelength scale. It provides a new paradigm for the design of optical components. At present, metasurface preparation technology can fabricate structures with sizes much smaller than the wavelength size of operating, which means that even if the structural elements are discrete, the operation of the wavefront can be considered continuous, which provides the possibility for a wide range of applications of metasurfaces, such as ultra-thin planar lenses [4], [5], wave plate [6], [7] and OAM operation [8], [9]. More importantly, metasurface has ultra-wideband achromatic capability in the visible light band via simple design, which significantly increases the application range of metasurface, such as meta-holography [10], [11] and optical image encryption [12], [13]. Thus, metasurface is proving to be a promising platform in optical display.

Compared with traditional optical display components, 3D display devices based on metasurface have obvious advantages [14], [15], [16], [17] in miniaturization, resolution, efficiency, and the field of view. For example, the most popular 3D near-eye displays use traditional stereoscopic methods to display 3D information. A significant problem with this approach is visual confusion and fatigue [18], and this phenomenon is called vergence accommodation conflict (VAC). To solve this problem, a metasurface with a small aperture (360 μm × 360 μm) is proposed as a display device to provide a virtual image without adjustment and projecting the image directly onto the retina [19]. On the other hand, high resolution and continuous depth are long-sought goals for 3D display components. To achieve this goal, a highly transparent Huygens’ metasurface is proposed to generate 3D images with a large (>10^8^) pixel count, subwavelength pixels, and continuous depth ranging from 0.5 to 2 m [20]. The field of view angle size is also an essential standard for yardsticking the performance of optical display devices. A kind of micro-LED based on gallium nitride metasurface is also proposed, which can display light information at arbitrary angles through reasonable design [21]. Optical metasurface has become an effective method to overcome the obstacles of traditional display technology. Therefore, using metasurface instead of conventional optical display elements is feasible.

This paper reviews the latest development of 3D display technology based on metasurface. After introducing the metasurface’s phase modulation method and structure resonance principle, we will further introduce metasurface hologram and color printing technology applied in future 3D display technology. The following section will describe the research progress of 3D display technology, analyzes the methods to solve the problems in traditional display devices from the perspective of geometric optics, and expounds on the advantages of 3D display devices based on metasurface from the standpoint of micro-nano optics. Finally, we will summarize the existing problems of metasurface used in 3D display platforms from the perspective of commercialization and look forward to the development direction of metasurface in the field of display, that is, the inverse design of AI-assisted metasurface and the absolute tunability and functional integration of metasurface performance using flexible or tunable material.

## 2 Physical mechanism of optical metasurface

With the deepening of the research on metasurface, the functions and classification of metasurface are diversified. However, the study on metasurface functions is inseparable from light modulation. Based on Huygens’ principle [22], every subwavelength meta-atom of a metasurface can be used as a point source in an arbitrary phase. Therefore, the phase modulation mechanism of the metasurface depends on the phase mutation caused by the scattering of meta-atoms, and the local phase of the space point can be changed by predesigning the geometric parameters of the meta-atoms to realize the wavefront control of the incident wave. For optical metasurface display components, typical phase modulation mechanisms include the geometric phase, propagation phase, resonant phase, and detour phase, and the spectral response mechanisms mainly include plasmon resonance and Mie resonance.

**Figure 1:**
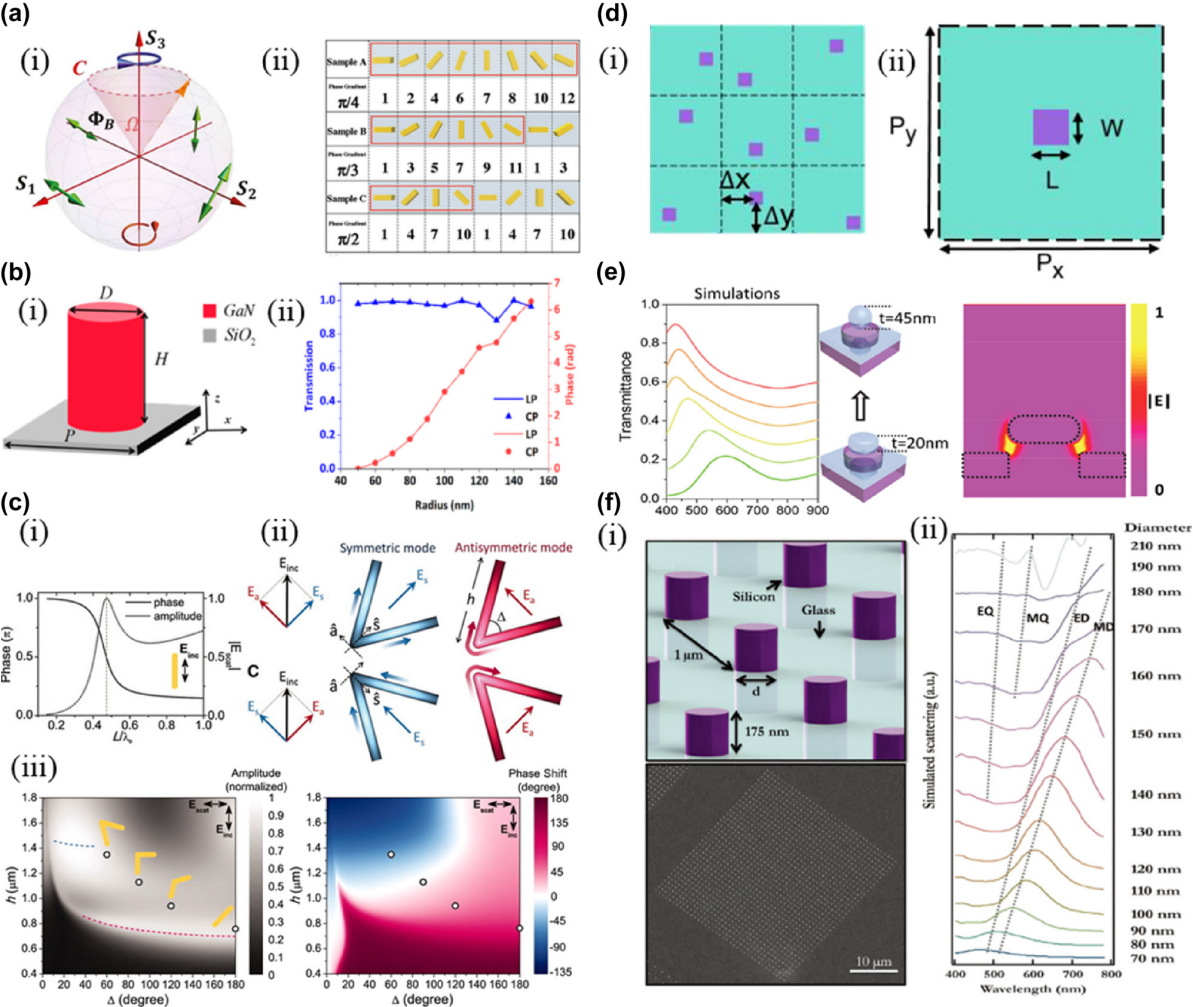
Physical mechanism of the optical metasurface. a) (i) Schematic illustration of the Poincare sphere [photonic spin Hall effect in metasurfaces: a brief review] and (ii) the nanorod-based metasurface with different geometric phase gradients [manipulation of the photonic spin Hall effect with high efficiency in gold-nanorod-based metasurfaces]. b) The GaN nanopillar composing metasurfaces and (ii) the correlation between the phase and diameter of the pillar. c) (i) Calculated phase and amplitude of straight rod antenna, (ii) A V-antenna supports symmetric and antisymmetric modes, and (iii) the amplitude and phase shift of the cross-polarized scattering light from a V-shaped antenna composed of circular gold rods with different lengths and angles. d) (i) Top view of the metasurface region and (ii) the unit cell. e) Simulated transmittance spectra for laser-modulated metasurfaces and normalized electric field distribution of the original plasmonic nanostructure. f) Schematic illustration of silicon nanopillar arrays and their SEM image and (ii) simulated scattering spectra using FDTD. The dotted lines indicate the evolution of peaks associated with the four fundamental modes (ED: electric dipole, MD: magnetic dipole, EQ: electric quadrupole, MQ: magnetic quadrupole). b) Reprinted from Ref. [25], under the terms of the Open Access Publishing Agreement; c) reprinted from Ref. [26], with permission from AAAS.

**Figure 2:**
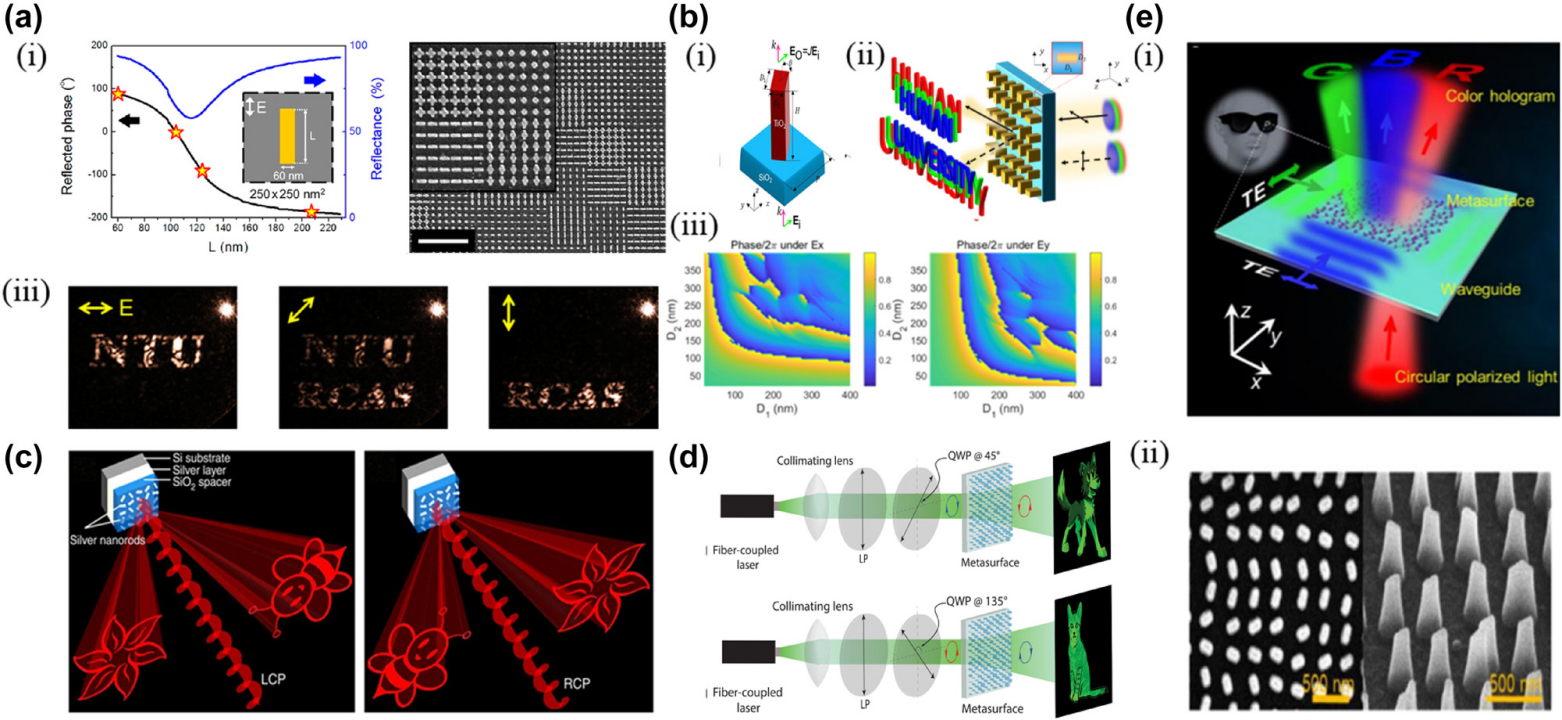
Polarization multiplexed meta-holography with linear and circular polarization channels .a) (i) Reflected phase and reflectance versus the length of Au nanorods, (ii) SEM image of the meta-hologram sample and the inset with higher magnification, and (iii) the reconstructed image under *x*-polarized, 45°-polarized, and *y*-polarized. b) (i) Schematic illustration of TiO_2_ meta-atom, (ii) two independent holographic images, “HUNAN” and “UNIVERSITY,” can be switched with two orthogonal polarization inputs, and (iii) the phase shift of the nanopillar under the *x*-polarization simulated and phase shift under the *y*-polarization obtained by transposition of the *x*-polarization results. c) Under the illumination of LCP light, the holographic images ‘flower’ and ‘bee’ are reconstructed on the left and right side, viewing from the direction of the incident light, respectively. The positions of the two holographic images are swapped when the helicity of incident light changes from LCP to RCP. d) A single metasurface encodes two independent hologram phase profiles for each circular polarization. When illuminated with RCP (LCP), the metasurface projects an image of a cartoon dog (cat) to the far field. e) (i) Conceptual illustration for three-polarization channel multiplexed meta-holography and (ii) SEM image of the fabricated sample. a) Reprinted from Ref. [27], with permission from AAAS; b) Reprinted from Ref. [28], under the terms of the Open Access Publishing Agreement; c) reprinted from Ref. [29], under the terms of the Open Access Publishing Agreement; d) reprinted from Ref. [30], with permission from American Physical Society; e) reprinted from Ref. [31], with permission from John Wiley and Sons.

The geometric phase originated in 1956, Pancharatnam [23] first proposed that cross-polarization scattering produces an additional phase factor exp(i2Δθ)exp(i2φ) when a plane structure is rotated counterclockwise by an angle relative to the *z*-axis. Then, in 1984, Berry [24] explained that the generation of this phase was due to the adiabatic evolution of the photon state in the high-latitude space, which was called the geometric phase, also known as the Pancharatnam–Berry (PB) phase. As shown in Figure 2a, the PB phase corresponds to the additional phase difference introduced in the path between two points on the Poincare sphere, equal to twice the rotation angle of each meta-atom. Therefore, phase modulation DOF of the geometric phase metasurface is the spatial orientation angle of the meta-atom, which can obtain efficient structural parameters by scanning the length and width of the meta-atom while rotating the meta-atom to obtain the required phase (Figure 1a). It can be seen that the geometric phase is only determined by the polarization state of the incident light and the rotation angle of the meta-atom, so the geometric metasurface has the characteristics of broadband response.

The metasurface composed of all-dielectric meta-atoms with a thickness close to the wavelength order is the propagation phase metasurface, whose phase modulation is based on the phase propagating to the entire meta-atom,

(1) $$\varphi =n_{\text{eff}}k_{0}d$$

where *n*_eff_ is the local effective refractive index of the meta-atom, *k*_0_ is the wavevector in a vacuum, and d is the thickness of the meta-atom. Therefore, the propagation phase can be achieved by modulating the period and structural parameters of each meta-atom [32]. In 2021, Sun et al. designed a high-efficiency metalens based on GaN according to the propagation phase design method [25]. The meta-atom diagram, transmission, and phase diagram are shown in Figure 1b.

Resonant phase metasurface was first proposed by Capasso’s research group at Harvard University in 2011, which uses local surface plasmon (LSP) resonance to modulate the phase of the antenna radiation field to achieve the modulation of the light field at the subwavelength scale [26]. As shown in Figure 1c, the discontinuous control of phase is realized by using a V-shaped antenna. A V-shaped antenna’s phase modulation degree of freedom (DOF) is arm’s length h and angle Δ, and high efficiency, covering 0–2π phase distribution of structural parameters can be obtained by scanning (Figure 1c). Metal-resonance metasurface typically employs V-shaped, Y-shaped [33], and C-shaped [34] to construct more complex meta-atoms. Due to the efficiency of metal metasurfaces and the limitations of the operating bands, the researchers proposed all-dielectric metasurfaces based on Mie resonance [35], [36], [37] and Fabry–Perot resonance [38], [39], [40], which has a thickness much smaller than the operating wavelength. Thus, it has higher working efficiency and lower loss.

The above-phase modulation method is generally suitable for the sub-wavelength scale, and the modulation ability of the optical diffraction effect is small or even non-existent. To make meta-atom modulate the phase of a particular diffraction order, the detour phase is proposed [31], [41], [42]. Detour phase modulation of *l* order diffracted light can be expressed as Δ*φ* = 2*πl*Δ*P*/*P*_0_, where Δ*P* is the lattice distance after displacement and *P*_0_ is the unit period of a meta-atom (Figure 1d). It can be seen that the detour phase is only related to the relative position of meta-atoms and has nothing to do with the frequency, polarization, and incidence angle. Simply by continuously changing the displacement of meta-atoms, the detour phase can be continuously adjusted from 0 to 2*π*.

Plasmon resonance occurs when the frequency of the incident light at the metal/dielectric interface matches the frequency of the electron oscillation on the metal surface. The electron density waves generated by plasmon resonance propagating along the surface are called surface plasmon polarization (SPP) waves [43] (Figure 1e). For an array with the period, the resonance wavelength can be approximated by the dispersion relation [43]:

(2) $$\lambda_{\text{SPR}}=\frac{P}{\sqrt{i^{2}+j^{2}}}\sqrt{\frac{\varepsilon_{\text{d}}\varepsilon_{\text{m}}}{\varepsilon_{\text{d}}+\varepsilon_{\text{m}}}}$$

where *i* and *j* are the scattering orders from the array. It can be seen from Equation (2) that the spectral response of the array can be adjusted by changing parameters such as period and aperture. Another mode of plasmon resonance is that under the excitation of incident light of a specific frequency, a collective oscillation such as field enhancement, strong light absorption, and scattering is generated on the metal surface, which is called localized surface plasmon resonance (LSPR) [44]. Unlike SPP, LSPR can localize the light wave energy in the subwavelength range to enhance the interaction between light and matter, and the dispersion condition must be met to excite SPP. At the same time, the excitation of LSPR does not. The resonance wavelength of LSPR is affected by the size, geometry, and properties of metal micro-nano structures. Because of the excitation of LSPR, metal metasurface can have obvious application value in the field of color printing [45], [46].

In dealing with the scattering problem of particles of comparable size and wavelength irradiated by plane waves in a uniform environment, Gustav Mie gave the solution of Maxwell’s equation, also known as the Mie solution [47], [48], and the scattering phenomenon is called Mie scattering. To accurately explain Mie scattering, Mie solution is used to analyze the scattered fields in the near and far field regions, as well as the total scattering cross section (*C*_sca_). *C*_sca_ can be expanded into an infinite series of spherical harmonic multipolar terms (Mie expansion):

(3) $$C_{\text{sca}}=\frac{2\pi }{k^{2}}\overset{\infty }{\underset{m}{\sum }}\left(2m+1\right)\left({\left|a_{m}\right|}^{2}+{\left|b_{m}\right|}^{2}\right)$$

where *a*_*m*_ and *b*_*m*_ are Mie coefficients of the *m*-order electric and magnetic multipole modes, respectively, and *k* is the wave number. Electric dipole (ED, *a*_1_^2^), magnetic dipole (MD, *b*_1_^2^), electric quadrupole (EQ, *a*_2_^2^), and magnetic quadrupole (MQ, *b*_2_^2^) are the first four multipole terms in Mie expansion. The scattering spectrum shown in Figure shows that the interaction between incident light and the dielectric resonator of meta-atoms generates ED, MD, EQ, and MQ fourth-order Mie resonance [49]. The resonant wavelength of each mode is different, resulting in a large color disk.

## 3 Modulation methods for the application of metasurface in the display field

To meet the human visual experience and transmit and reproduce real optical information, pursuing high-performance and multi-functional image and color display has become one of the goals in the display field. Inspired by the bright colors shown by the interaction between micro and nanostructures and light on the body surface of some animals and plants in nature, scientists started the research of micro and nanostructure display components. In recent years, with the rapid development of micro-nano machining technology, the ability of metasurface to modulate wavefront and spectrum to realize structure color and holographic display has become an important research field in nanophotonics. However, to further improve the color quality and the meta-hologram’s information capacity, the metasurface for the multiplexing of holographic images and generating high-quality structural colors has been studied and explored continuously.

### 3.1 Multiplex metasurface holography

In the field of holography multiplexing, metasurface has shown a strong ability. Firstly, a polarization multiplexing metasurface is proposed according to the different polarization of light to realize the response to different images. Here, 12 polarization multiplexing channels are obtained [50], showing powerful dynamic vector holographic display and encryption functions. In addition, other depth-of-modulation (DOMs) of super-holographic multiplexing include incidence angle [51], [52], wavelength [53], [54], [55], [56], and OAM [57], [58], [59], [60], [61], [62].

#### 3.1.1 Polarization multiplexing

Polarization multiplexing, as one of the most important multiplexing methods for metasurfaces, has the characteristics of high efficiency and low crosstalk. At present, there are two ways to realize metasurface polarization multiplexing. One is to use the birefringence characteristic of the meta-atom to modulate the phase of different polarized light independently. The other is to interlace the meta-atoms corresponding to different polarizations onto a metasurface to achieve phase modulation for different polarization incident lights. Chen et al. used the resonance phase of the long axis of the gold nanorods in response to linearly polarized light to superposition two gold nanorods into a cross-shaped meta-atom. They designed a metasurface capable of producing different holograms in different polarization directions [27] (Figure 2a). The cross-shaped structure only takes advantage of the response of the birefringent microstructure in one direction; that is, the resonance properties of the single rod are more sensitive to the long axis than the short axis [28]. If birefringent microstructures simultaneously satisfy the requirements of two orthogonal polarization phases, the design complexity of the metasurface can be further simplified. Figure 2b shows a typical example of the use of this method, where the anisotropic metasurface consists of birefringent titanium dioxide (TiO_2_) nanorods. The phase delay of incident *x* and *y*-polarized light can be independently modulated by a reasonable selection of the structural parameters of the nanorods, achieving seven-channel metasurface holography [63]. Very recently, two landmark studies have pushed the capacity of polarization multiplexing far beyond the conventional three-channel limit. Xiong et al. introduced an engineered-noise strategy that enables 11 fully independent polarization channels in a single dielectric metasurface hologram, achieving a channel-to-channel isolation and demonstrating complex keyboard-style image sets [64]. Building on this concept, Wang et al. exploited non-orthogonal polarization bases and a vectorial diffraction neural network to reconstruct 55 low-crosstalk holograms from one metasurface, effectively expanding the Jones matrix to a 10 × 10 dimensionality and pointing to a scalable route toward ultra-high-capacity information encoding [65].

The multiplexing of circularly polarized light is generally based on the polarimetric properties of geometric phases. The meta-atoms corresponding to left and right circularly polarized light are interleaved to realize circularly polarized multiplex metasurface holography. Wen et al. demonstrate a geometric phase metasurface hologram based on Meta-Insulator Metal (MIM), in which the position of both holograms become interchangeable by controlling the helicity of incident circularly polarized light [29] (Figure 2c). More flexible circular polarization multiplexing and larger capacity multi-polarization multiplexing metasurface holography can be achieved by combining geometric phase and other phase modulation methods (e.g., propagating phase, detour phase). Mueller et al. combined the geometric phase and the propagation phase to realize the independent control of the phase of any orthogonal polarization state [30]. As a verification, two holograms can be obtained by incident LCP and RCP, respectively (Figure 2d). In addition, multi-polarization multiplexing metasurfaces increase the multifunctionalization in the field of meta-holography by combining the geometric phase and the detour phase. Shi et al. proposed on-chip metasurface holography multiplexing using this phase combination method [31]. The detour phase adds additional DOM to the phase modulation, and different holograms can be generated under the incident of different directions of TE waves and circularly polarized light (Figure 2e).

#### 3.1.2 Angle multiplexing

**Figure 3:**
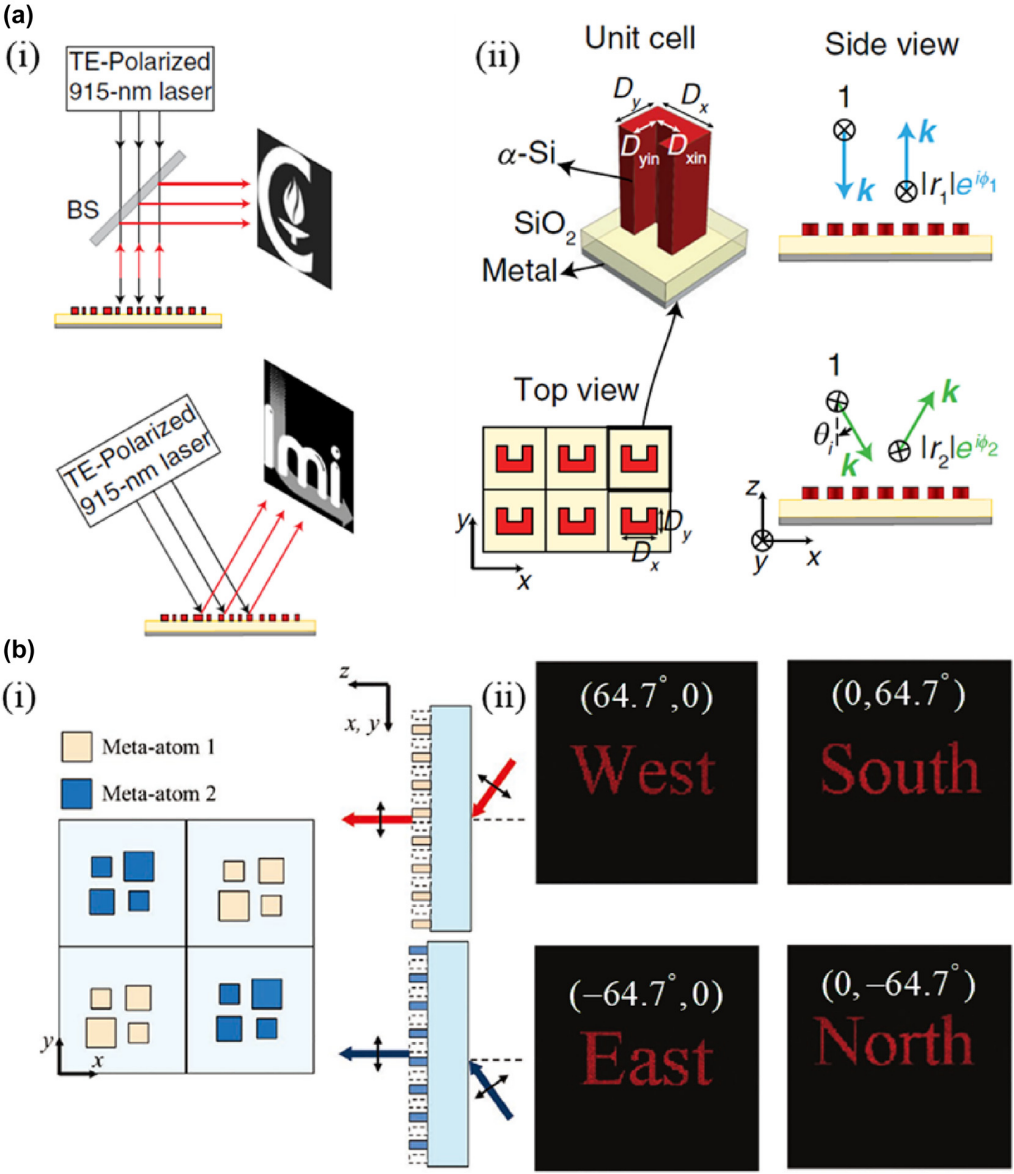
Angle-multiplexed meta-holography. a) (i) Simplified drawing of the measurement setups under normal and 30° illumination angles and (ii) Schematic illustration of various views of a uniform array of U-shaped cross-section α-Si meta-atoms arranged in a square lattice resting on a thin SiO_2_ spacer layer on a reflective surface (i.e., a metallic mirror). The array provides an angle-dependent response such that TE-polarized light at 0° and 30° illumination angles undergoes different phase shifts as it reflects from the array. b) (i) Top view of a composite composed of meta-atoms 1 and 2 and their diffraction characteristics depending on the incident angles and (ii) the reconstructed image according to the angle of incidence (*θ*_*x*_, *θ*_*y*_). a) Reprinted from Ref. [51], under the terms of the Open Access Publishing Agreement; b) reprinted from Ref. [52], with permission (CC BY 4.0).

Generally speaking, the wavefront modulation function of the metasurface has little dependence on the angle of incidence [66], [67], which results in the angle of incidence having a fixed response over a large range [68]. Still, distortion and efficiency reduction may occur at non-design angles of incidence [66], [69], [70]. Kamali et al. proposed an angle-multiplexed metasurface for independent wavefront coding at different incident angles to solve these problems [51]. The U-shaped meta-atoms that make up the metasurface provide the ability to independently modulate the reflection phase at two different incident angles, breaking the basic optical memory effect of the metasurface. At 0° and 30°of incidence, two different holographic images can be observed (Figure 3a). To meet the requirements of phase modulation at different incident angles, many U-shaped meta-atoms with different structural parameters are needed to compose the metasurface.

**Figure 4:**
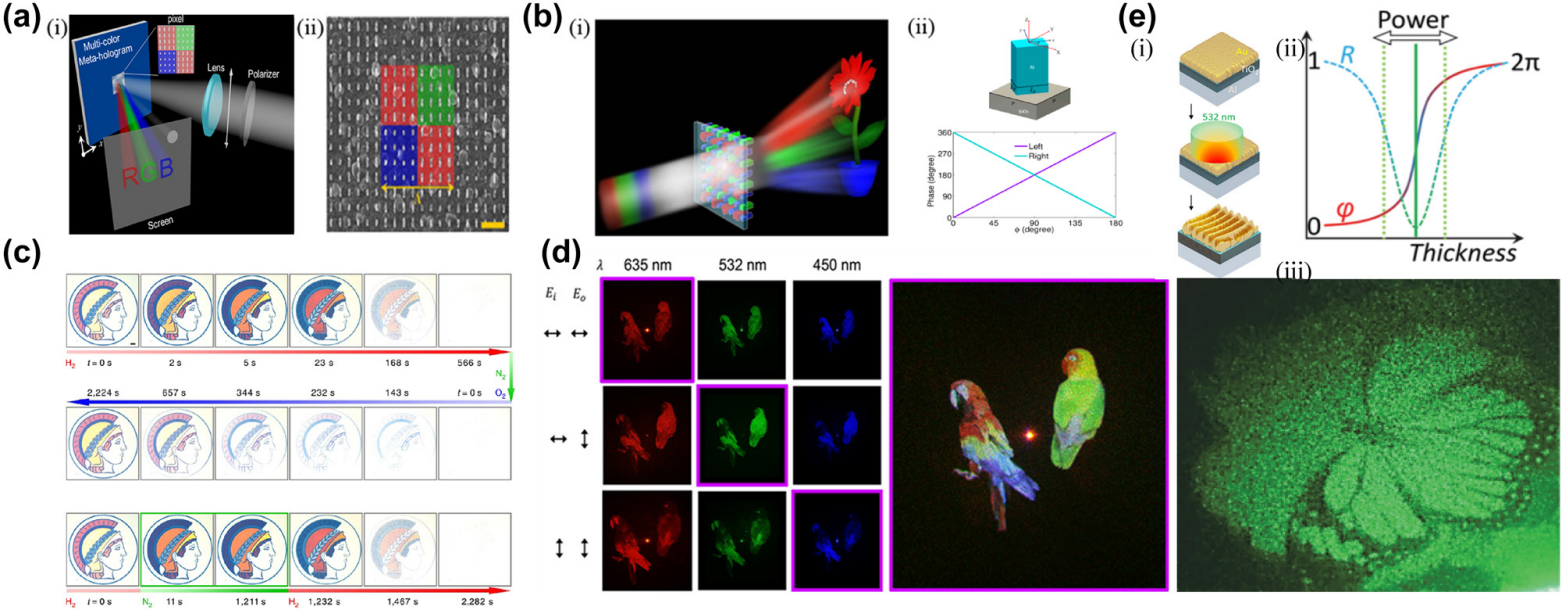
Multicolor and reconfigurable meta-holography. a) (i) Illustration of the designed multicolor meta-hologram (MCMH) under linearly polarized illumination and (ii) SEM image of the MCMH sample; the colored corresponds to a single meta-atom comprising four sub-units. b) (i) Schematic of a metasurface-generated multicolor hologram and (ii) schematic of a meta-atom and the phase shift from a meta-atom at different incident angles. c) Optical micrographs of the Minerva logo recorded during cyclic hydrogenation and de-hydrogenation; the intermediate frozen state obtained by removing the hydrogen supply. d) Holographic reconstructions obtained using the complete set of wavelength and polarisation multiplexed channels; the channels highlighted by the purple box are those employed for full-color holography. e) (i) Schematics of the FP-type hybrid metasurface with Au–TiO_2_-AI coatings. (ii) Schematic of laser printing power modulateing the effective film thickness, decoupling amplitude and phase at the critical transition point, (iii) laser printed hologram of a butterfly. a) Reprinted from Ref. [71], with permission from ACS; b) reprinted from Ref. [72], with permission from ACS; c) reprinted from Ref. [53], under the terms of the Open Access Publishing Agreement; d) reprinted from Ref. [63], with permission from ACS; a) reprinted from Ref. [73], with permission from ACS.

The above methods are insufficient for the large capacity requirements of meta-holography, and the angle multiplexing methods also have limitations. Therefore, Jang et al. proposed an angle multiplexing metasurface holography with a detour phase that does not require many different meta-atoms [52]. Figure 3b shows the functional diagram of the metasurface, which can realize four-channel angle-multiplexed meta-holography according to the detour phase. The composite pixel of the metasurface consists of two meta-atoms, the beige meta-atom one diffracting the x and y positive semi-axis (*θ*_*x*_ > 0 or *θ*_*y*_ > 0) incident light to the vertical direction, and the blue atom diffracting the *x* and *y* negative semi-axis (*θ*_*x*_ < 0 or *θ*_*y*_ < 0) incident light to the vertical direction. Thus, the metasurface can generate four independent holograms (Figure 4b).

#### 3.1.3 Wavelength multiplexing

Wavelength multiplexed metasurfaces, such as achromatic metalens [74], are widely applied. For meta-holography, wavelength multiplexing is mainly used for colorful holographic imaging. Among them, the most direct wavelength multiplexing method interlaces the atoms that respond to different wavelengths. Huang et al. implemented a reflective polychromatic hologram (Figure 4a) using an interlace aluminum nanoarray, in which one meta-atom of the metasurface consists of three subunits [71]. Each subunit has high reflectivity only for a specific wavelength range and exhibits absorption properties at other wavelengths. The same reflectivity for three different wavelengths of incident light is ensured by reasonably designing the size of the meta-atom. Full-color holography is also realized using this method [56]. Wang et al. proposed a transmission metasurface composed of rectangular silicon nanopillars capable of independent phase modulation at red, green, and blue (RGB) wavelengths; the metasurface has an achromatic function and displays highly dispersive colorful hologram [72] (Figure 4b).

The phase compensation scheme using the dispersion of the structure itself at different wavelengths is also proposed in addition to the staggered arrangement of multiple meta-atoms. It is necessary to code the independent phase of a single meta-atom at multiple wavelengths to achieve wavelength multiplexing [72], [75]. In the phase modulation method of the metasurface, only the geometric phase is achromatic. Still, the wavelength is a variable for the phase of the metalens, resulting in the achromatic capability of the geometric phase metalens not being realized. The researchers used the propagation phase dispersion of the meta-atom to continuously compensate for the chromatic aberration of the geometric phase metalens and realized the achromatic metalens in visible [76], [77], near-infrared [54], [78], [79], mid-infrared [80], [81], and terahertz bands [82]. Inversely, the method can also increase the chromatic aberration of the lens to realize the metachromatic device [83], [84] that can be used for spectral analysis. It is worth noting that if the continuous compensation in the scheme is discretized, the wavelength multiplexing function of the metasurface can be realized by a specific design at a single wavelength [85]. Shi et al. used this method to implement OAM multiplexing at different wavelengths [16]. The above methods promote the development of metasurface holography and provide a solution for flexibly producing high-performance full-color holograms. Beyond wavelength-selective meta-atom interleaving, a new generation of inverse-designed metasurfaces is rapidly redefining color holography. So et al. employed gradient-descent optimisation to encode nine multiplane RGB images and the first metasurface-based 3D hologram into a single phase-only device, achieving record information density [86]. Building on this idea, Yin et al. introduced an end-to-end inverse-design framework that delivers 12 channels spanning wavelength, depth and polarisation with crosstalk-free reconstruction, all realised with meta-atoms offering only two geometric degrees of freedom [87]. Most recently, Chi et al. integrated a dispersive full-Jones-matrix neural network into a global optimiser, enabling tri-polarisation, multi-wavelength, multi-depth holography and a six-fold SSIM improvement over separated designs [88]. These inverse-design advances complement the passive-dispersion strategies discussed above and point to a scalable route toward ultra-compact, high-fidelity full-color holographic displays. Multiplexing different color information on different meta-atoms can result in a dynamic color display [53] (Figure 4c) and high-performance full-color holograms with multi-channel polarization wavelength multiplexing [63] (Figure 4d).

Zhu et al. newly proposed resonant laser printing (RLP) technology utilizes the resonance absorption generated by lasers in optical cavities formed by multilayer metals and dielectric coatings. This enables the printing of nano-scale structures, which is instrumental in fabricating optical metasurfaces. This development offers a novel approach for the preparation of color holograms [73].

#### 3.1.4 OAM multiplexing

**Figure 5:**
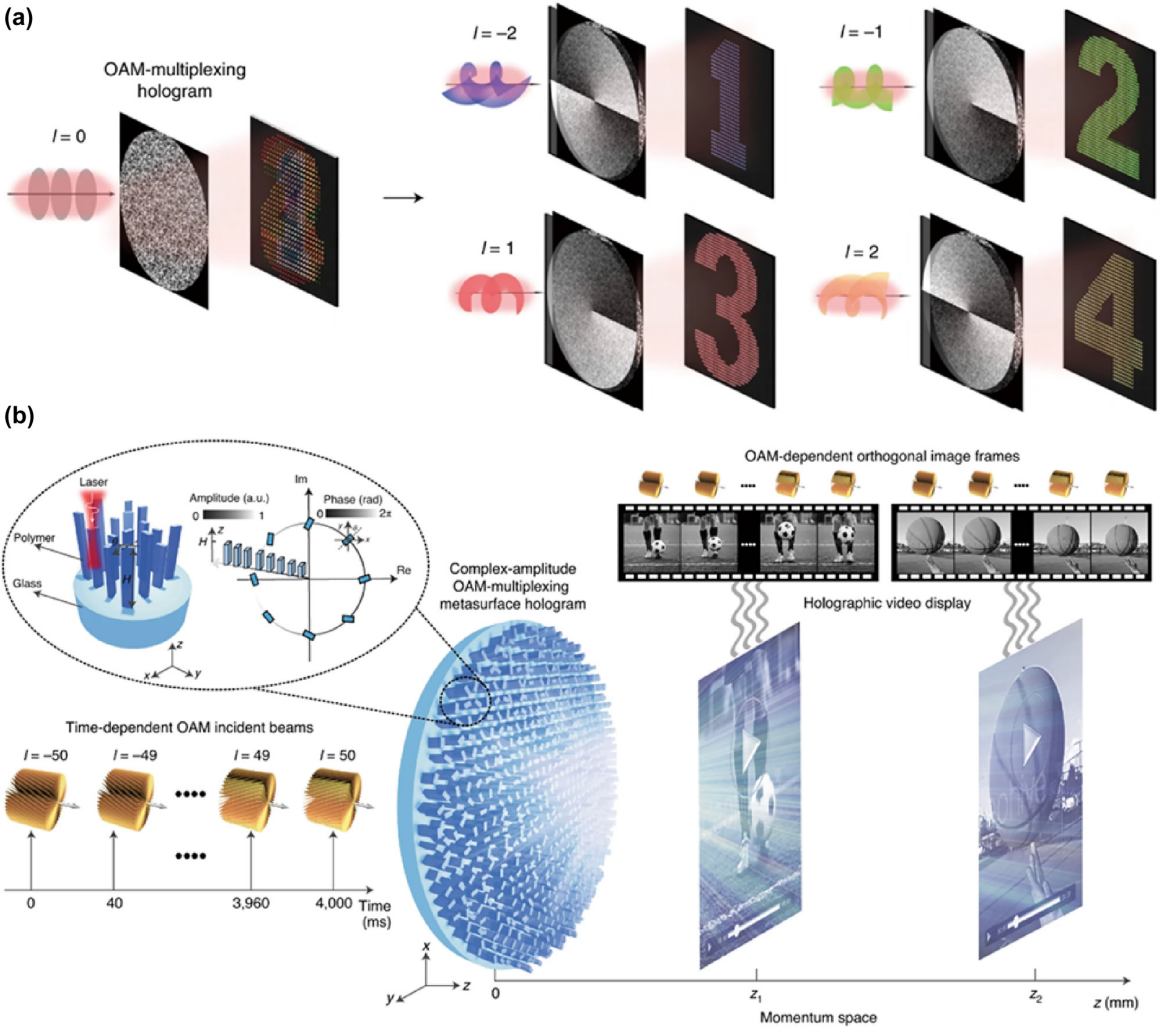
Orbital angular momentum (OAM) multiplexed meta-holography. a) Schematic of an OAM-multiplexing hologram capable of reconstructing OAM-dependent holographic images. b) Schematic of complex-amplitude metasurface-based OAM holography that encodes 200 OAM-dependent holographic images and can make holographic videos. a) Reprinted from Ref. [62], with permission from Springer Nature; b) reprinted from Ref. [61], with permission from Springer Nature.

Information can be transmitted independently using the spatial dimension of the OAM beam as the information carrier. In 1992, Allen et al. experimentally concluded that OAM is carried by wavefront with a helical phase [89], the phase factor is exp(*ilφ*), *l* is the topological charge, *φ* is the azimuthal angle. Because of the orthogonality between the number of spiral modes and the different orders, OAM has become a kind of optical DOF that attracts much attention [89], [90], [91], [92], [93], [94], which is used to improve the optical and quantum information capacity. The combination of OAM holography and high-resolution metasurface to achieve digital holography has become a research hotspot, opening up a new path for ultra-high-capacity holographic devices. Such Fang et al. superimposed the phases corresponding to different *l* to construct an OAM multiplexed phase-modulated metasurface [62] (Figure 5a). Here *l* = 2, 1, −1, −2, there are four independent holograms when OAM modes corresponding to different *l* incidents are on the metasurface. This phase-only modulation method increases the crosstalk between different holograms. It is not easy to increase the number of holographic channels due to the limitation of the degree of freedom of nanosized meta-atoms. Ren et al. proposed an OAM multiplexed complex amplitude-modulated meta-holography [61] for this case. Complex amplitude modulation is achieved by changing the element atoms’ height, which is uncommon. Moreover, a random phase function reduces the amplitude variation and eliminates the coherence between different holographic channels. In the ultra-wide range of topological charges of −50 to 50, the metasurface can generate up to 200 different holographic images, making holographic videos (Figure 5b), demonstrating the superpower of OAM multiplexing as an information carrier. A further leap was achieved by Meng et al., who coupled orbital angular momentum with ultranarrow-linewidth wavelength selectivity, enabling a transformer-designed metasurface to reconstruct 118 independent images for wavelength-vortex multiplexed holography [95].

#### 3.1.5 Superimposed multiplex metasurface

**Figure 6:**
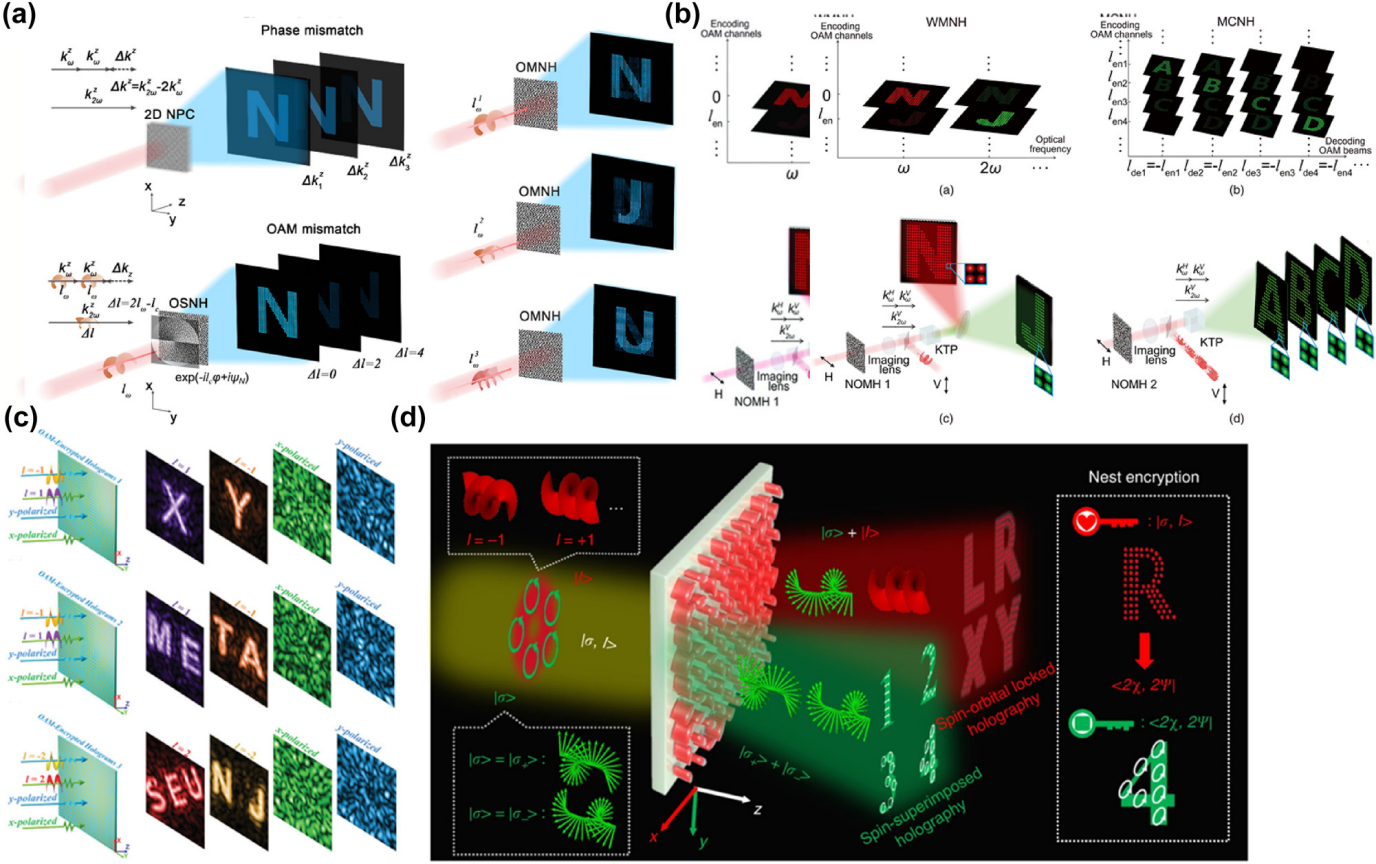
Nonlinear and angular momentum multiplexed holography. a) Conceptual illustrations for Fourier-domain nonlinear OAM matching and multichannel nonlinear holography. b) Principle of OAM multiplexing nonlinear holography and its applications in type-II SHG. c) Schemes of OAM-encrypted holography. d) The angular momentum holography depends on arbitrary superimposing of the SAM and OAM eigenstates in the output field. a) Reprinted from Ref. [100], under the terms of the Open Access Publishing Agreement; b) Reprinted from Ref. [100], under the terms of the Open Access Publishing Agreement; c) Reprinted from Ref. [101], with permission from John Wiley and Sons; d) reprinted from Ref. [102], under the terms of the Open Access Publishing Agreement.

To improve the security of multi-DOF OAM multiplexed holography, combining OAM with other dimensions can improve the reliability of information security and increase multiplexing channels. With the study of nonlinear crystals, holography has been extended to nonlinear fields [96]. By designing seven nonlinear crystals, arbitrary harmonics can be manipulated [97]–[99] to achieve simultaneous wavelength and OAM multiplexing. Fang et al. used OAM with different topological charges to encode holograms, which can realize the switching of holograms in the second harmonics and the nonlinear holographic images based on OAM [100] (Figure 6a). Subsequently, Fang et al. improved this method and utilized OAM to encode multiple images at a single wavelength, increasing the number of multiplexed channels [100] (Figure 6b).

Polarization encryption OAM multiplexing holography realizes OAM multiplexing under different polarization channels and combines polarization with OAM to realize more channel numbers. Xiao et al. have conducted in-depth research on metasurfaces and designed two metasurfaces, one for generating vortex light and the other for realizing polarimetric encrypted OAM multiplexed holography. The preset image can only appear when the OAM beam carrying reverse topological charge is irradiated at the incident of the preset polarization state [101]. As shown in Figure 6c, a disordered image will appear when incident *x*-polarized or *y*-polarized light is incident, and a preset hologram will appear only when the topological charge number of *x*- and *y*-polarized light is *l* = 1 and *l* = −1, respectively.

Spin angular momentum (SAM) usually appears as circular polarization. Yang et al. proposed that angular momentum multiplexing holography can realize arbitrary SAM and OAM dimensions adjustment in sub-wavelength scales [102]. As shown in Figure 6d, the preset hologram can be obtained only when the beam carrying specific SAM and OAM eigenstates is illuminated to the metasurface, and the superposition of the two SAM eigenstates can realize any polarization states, thus realizing OAM multiplexed super-holography with arbitrary polarization encryption.

### 3.2 Metasurface coloring for display technology

In addition to the application of metasurface wavefront modulation in holographic projection, metalens, beam refraction, etc., another application is displayed, which is the use of structural resonance modulation to generate structural color. The generation of structural color is essentially the color effect resulting in scattering, reflection, diffraction, and interference of light in the micro-nano structure without the need for any dye, which shows the advantages of high resolution, high contrast, high stability, low power consumption, and recyclability [103], [104], [105], [106]. According to the coloring formation mechanism of the structure color, the structure color is mainly divided into the structure color based on the plasma resonance effect of metal materials and the structure color based on the Mie resonance effect of dielectric materials. Relying on relevant published papers, these two structural colors will be elaborated below.

#### 3.2.1 Plasmonic metasurface structure color

**Figure 7:**
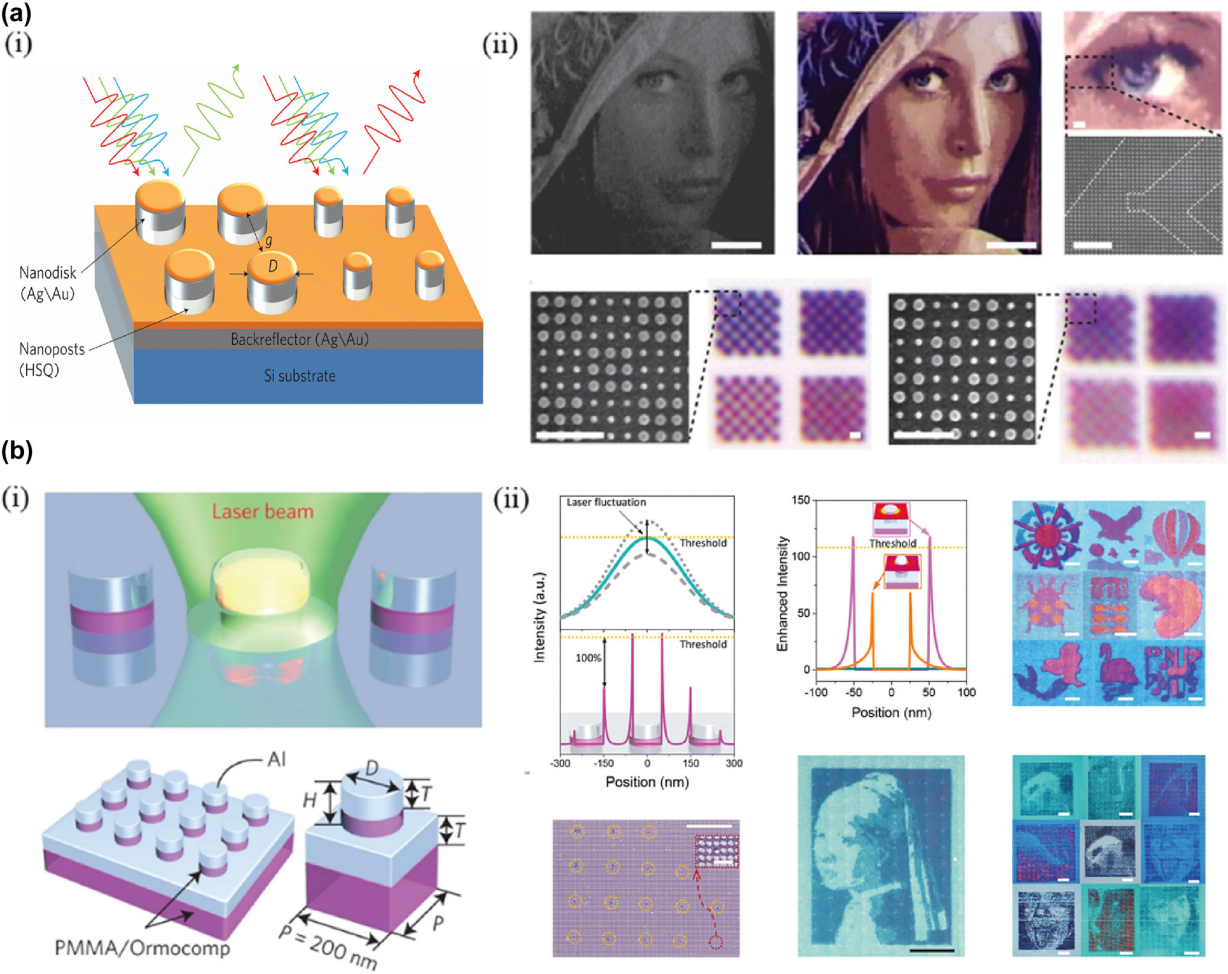
Metasurface-based color printing and laser printing. a) (i) Schematic illustration of a metasurface, as a result of the different diameters (D) and separations (g) of the nanodisks within each pixel, different wavelengths of light are preferentially reflected back and (ii) full-color image printing and resolution test patterns. b) (i) Schematic illustration of laser printing and schematic illustration of the plasmonic metasurface and (ii) laser printing with sub-diffraction-limit resolution. Printed images in different color schemes with single-unit-cell resolution. The laser energy used for patterning was moderated to minimize the influence on different printing channels, whereas strong power will incite crosstalks of plasmonic energy distribution between the neighboring unit cells and degrade. a) Reprinted from Ref. [107], with permission from Springer Nature; b) reprinted from Ref. [114], with permission from Springer Nature.

The method of generating structural color from the metasurface begins with the plasma resonance response of metal nanostructures. Using plasma scattered light, Kumar et al. proposed a method capable of producing ultra-high resolution color-printed images of ∼100,000 points per inch (DPI) close to the light diffraction limit [107] (Figure 7a). However, plasma color image printing requires focused ion beam (FIB) [108], [109], or electron beam lithography (EBL) [110], [111], [112], [113], both of which are inflexible, expensive, and unsuitable for dynamic customization. In this regard, Zhu et al. proposed a resonant laser printing (RLP) technique for color printing on nano-imprinted plasma metasurface using laser post-write technology. The basis of RLP is that strong on-resonant energy absorption under pulsed laser radiation causes the lattice temperature to increase locally in an ultra-short time (1 ns), leading to rapid melting, which changes the morphology of the structure surface and modifies the structure color. When using nanosecond laser pulses, the aluminum (Al) disc transforms into a sphere and eventually ablates. In this process, the resonance absorption wavelength is blue-shifted, and the structural color is also changed from blue to yellow. Using this technique, color-printed images with a resolution of up to 127,000 DPI can be produced at low power with 0.3 nJ per pixel [114] (Figure 7b). However, plasma resonance results in the metasurface having a large bandwidth absorption, thus reducing the saturation of the structural color. Zhu et al. subsequently proposed the digital resonant laser printing (DRLP) technology [115] and a complete solution for manufacturing large-scale low-cost plasma-colored metasurface [116], a flexible post-fabrication writing technique that can be used for large-scale customization of optical metasurfaces. By modifying the amplitude, phase, and polarization of reflected and transmitted light, it alters the optical properties of the metasurfaces. This technology offers a promising approach for low-cost customization of photonic devices with subwavelength elements.

#### 3.2.2 Dielectric metasurface structure color

Metasurface structure color based on dielectric material compensates for the optical loss of plasma structure color and has been widely investigated. Dielectric metasurface structure color uses the Mie resonance that depends on the geometry and size of the meta-atoms [117], [118], [119], [120], [121], [122], [123]. In principle, dielectric meta-atoms with a high refractive index *n* can modulate electric dipoles and magnetic dipoles, but plasma metasurfaces mainly use the mechanism of modulating electric dipoles. Mie scattering occurs when the wavelength *λ* of the incident light is close to the size *R* of the meta-atom (2*R* ≈ *λ*/*n*), and this mechanism provides the opportunity to achieve dielectric metasurface structure colors using higher-order multipoles generated by Mie resonances [124].

Dielectric metasurfaces generate structural colors, the most commonly used materials for which are amorphous silicon (a-Si) [125], [126], [127], crystalline silicon (c-Si) [128], [129], and titanium dioxide (TiO_2_) [130], [131]. Due to the high refractive index of the silicon metasurface, it exhibits a strong electric magnetic resonance after interacting with visible light. Using this property, silicon is prepared into different meta-atom shapes, such as nanopillar [132] and cross [133], resulting in high purity and wide color gamut colors.

**Figure 8:**
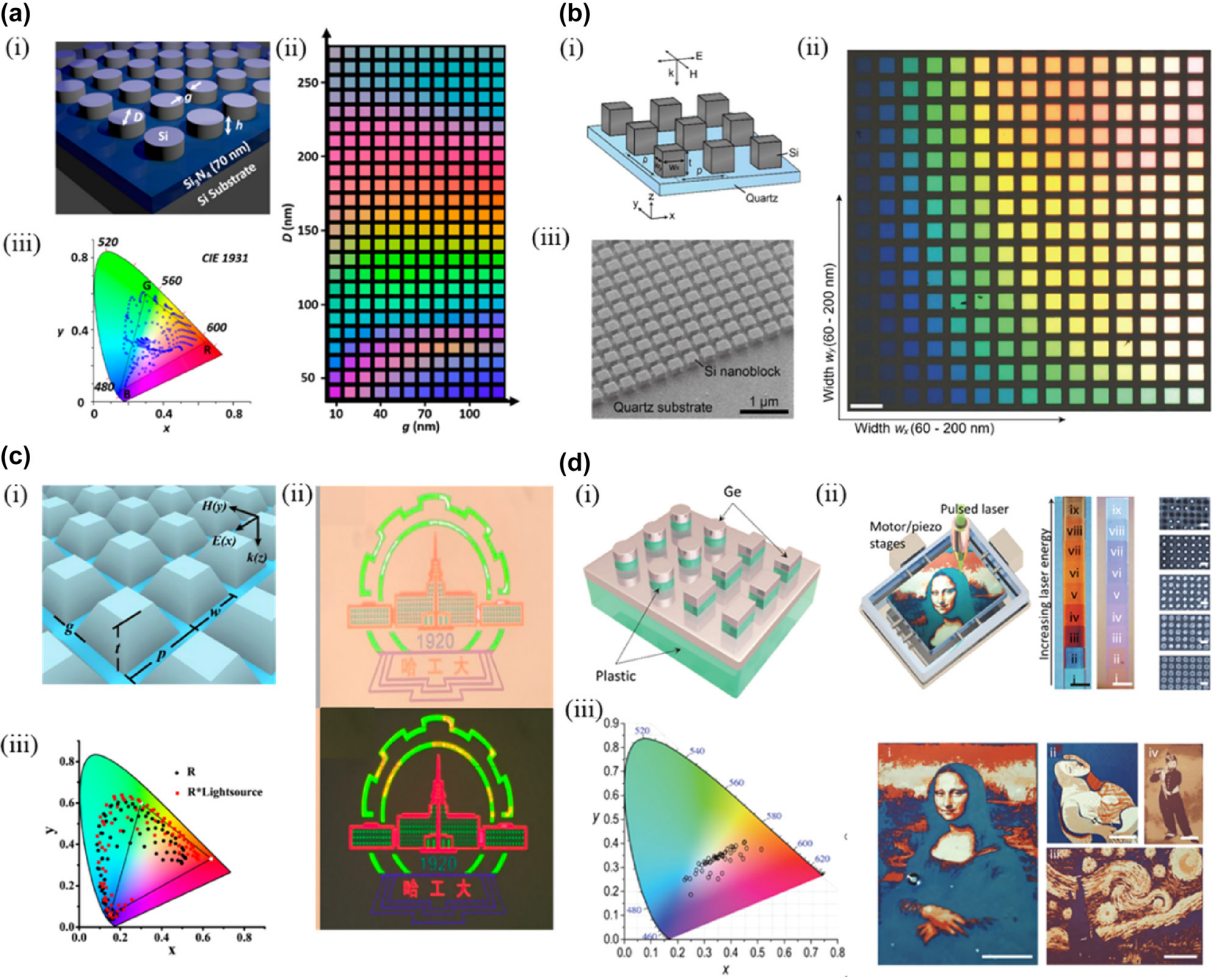
Structural color generation using dielectric metasurfaces. a) (i) Schematic of the color pixel design consisting of silicon nanodisks/70 nm thick Si_3_N_4_/silicon substrate, (ii) bright-field optical micrographs of the basic silicon color palette and (iii) CIE *xy* chromaticity coordinates of measured reflectance spectra of color palette occupying ∼120 % of the sRGB gamut. b) (i) Schematic of the Si nano block array on a quartz substrate, (ii) Optical microscope image of the fabricated all-dielectric nano block arrays through a 20× objective (NA: 0.45) irradiated with ex-polarized white light, and (iii) oblique SIM image of the Si nano block array. c) (i) The tilt-view and parameters of the TiO_2_-based metasurface, (ii) the corresponding reflection colorful images of the university logo under a bright field microscope and the polarizing microscopy image of the logo, and (iii) the directly calculated (black dots) and corrected (red squares) structural colors from the simulated reflection spectra of metasurfaces with varying dimensions in CIE 1931 color map. d) (i) Schematic illustration of the metasurface, (ii) schematic setup of RLP and laser-printed paintings, and (iii) structural color gamut of measured reflectance spectra presented in a standard CIE-1931 color space. a) Reprinted from Ref. [127], with permission from ACS; b) reprinted from Ref. [129], with permission from ACS; c) reprinted from Ref. [130], with permission from ACS; d) reprinted from Ref. [134], under the terms of the Open Access Publishing Agreement.

For a-Si metasurface, the substrate effect limits the saturation and gamut of structural colors, resulting in higher loss. Substrainless meta-atoms can suppress light scattering and exhibit a narrower spectrum. Thus, Dong et al. proposed a new metasurface design while maintaining the resolution [127]; as shown in Figure 8a, a-Si nanopillars are covered on Si_3_N_4_ film with an a-Si substrate at the bottom. Si_3_N_4_ acts as an antireflective layer to inhibit the substrate effect, and the Kerker effect sharpens the reflection peak. The structural color generated by the metasurface has high saturation, and the sRGB color gamut range reaches 120 %. Compared to a-Si, c-Si has a lower loss in visible light. Takahara et al. prepared nanopillars using c-Si and generated structural colors by adjusting the geometric parameters of the nanopillars [129] (Figure 8b). The resolution of the structural color can reach 85,000 DPI under the premise of achieving high saturation.

Although the Si metasurface achieves high saturation structure colors, the constant loss of Si materials still hinders the generation of higher saturation structure colors. As a candidate material for future all-dielectric coloring components, TiO_2_ has an extinction coefficient of almost 0 in the visible range. Sun et al. used a photonic bandgap formed by TiO_2_ meta-atoms to generate structural colors with high brightness and saturation [130] (Figure 8c).

The above dielectric metasurface is a static coloring technology with limitations. The RLP technique proposed by Zhu et al. to generate dynamic structural color can also be applied to all-dielectric nanostructures. For instance, Zhu et al. designed a Germanium (Ge) metasurface to achieve dynamic color rendering using the RLP technique [134] (Figure 8d). In addition, metasurface dynamic structure color generation methods are chemical reaction [53], [135], [136], liquid crystal [137], [138], [139], polarization dependent [140], [141], [142], electrical stimulation [143], [144], [145], and mechanical deformation [146], [147], [148], [149].

### 3.3 Bifunctional metasurface

**Figure 9:**
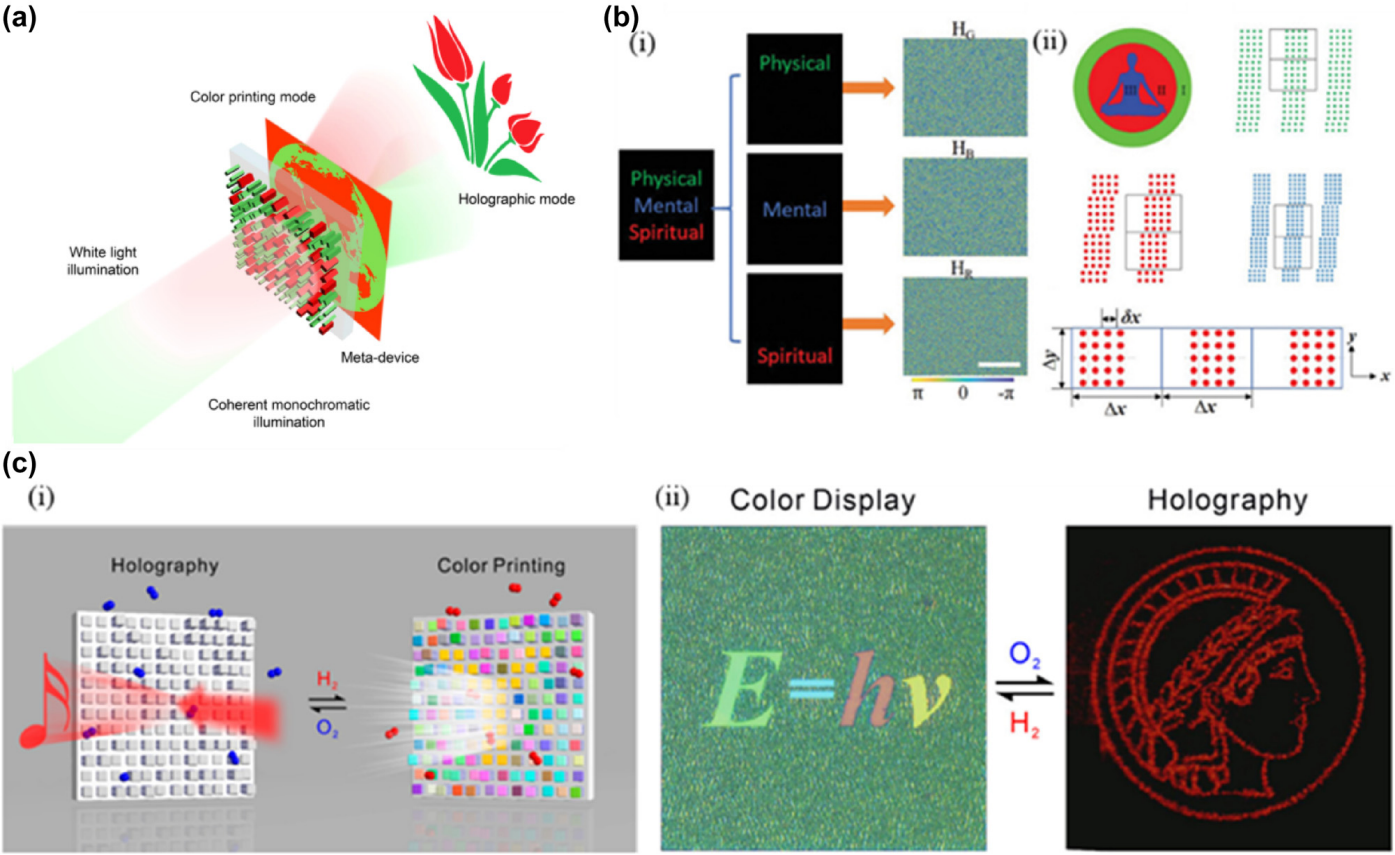
All-Dielectric Metasurface with Dual-Functionality for Color Printing and Far-Field Holography. a) Schematic illustration of the all-dielectric metasurface that integrates dual working modes for incoherent color printing and far-field holography by simultaneously modulating spatial and spectral responses. b) The design principle encodes a color hologram into a color-printed image. (i) The target holographic image consists of the words “Physical” (in green), “Mental” (in blue), and “Spiritual” (in red). The Target image is decomposed by color into three sub-images, and three phase-only holograms HG, HB, and HR are calculated, and (ii) the Yoga is image to be color-printed and schematic illustrations of green, red, and blue supercells. A black box indicates the extent of each supercell. c) (i) Schematic illustration of the dual-function switching between dynamic holography and dynamic color display by hydrogenation (H_2_) and dehydrogenation (O_2_) and (ii) switching between dynamic color display and dynamic holography through hydrogenation and dehydrogenation. a) Reprinted from Ref. [150], under the terms of the Open Access Publishing Agreement; b) reprinted from Ref. [151], with permission from John Wiley and Sons; c) reprinted from Ref. [152], under the terms of the Open Access Publishing Agreement;

Metasurface structure colors and color holograms are often produced by different design mechanisms, raising whether the two can be combined into a single component. However, most of the reported metasurface structure colors do not have the function of encoding phase distribution and depth information, so holograms cannot be reconstructed. Similarly, most holograms are not designed to modulate the spectral response of light, which typically behaves in a random pattern under incoherent light. To combine the two functions, the metasurface must modulate the spatial phase and the spectral response. Huang et al. integrated color printing and computer-generated holograms in a single-layer a-Si metasurface [150]. The metasurface is composed of two types of meta-atoms, which modulate the geometric phase of the red and green laser, respectively, and a “flower” hologram can be obtained in the far field; simultaneously, a color image of the “Earth” can be displayed under white light irradiation (Figure 9a). In addition to bifunctional metasurfaces based on silicon, Crozier et al. prepared a TiO_2_-based bifunctional metasurface [151]. The required reflection spectrum and color hologram are obtained by changing the size of TiO_2_ meta-atoms to support the Mie resonance of the visible wavelength. The “yoga” image can be displayed directly under white light irradiation (Figure 9b). The off-axis design is used to avoid crosstalk between the holographic image and the zero-order point when the red, green, and blue laser is illuminated on the metasurface; color word images can be seen (Figure 9b).

However, the above single-layer bifunctional metasurface has some defects, such as low efficiency, polarization sensitivity, and static modulation, which cannot meet the requirements of dynamic display. Liu et al. realized the dual function switching between dynamic holographic display and dynamic color printing by utilizing the accompanying optical response changes during the interconversion process of metal and dielectric [152]. The transformation property of magnesium (Mg) into magnesium hydroxide (MgH_2_) by hydrogen absorption gives the metasurface a dynamic function. The incident light experiences multiple reflections in the Fabry–Perot nanocavity, and spectral response occurs. The phase difference becomes very small at this time, so the holographic display function is hidden. When placed in an oxygen environment, MgH_2_ can be restored to Mg. The metal Mg coating can effectively reflect the incident light in the visible light range, realizing the function of holographic display, and the structural color function is erased (Figure 9c).

## 4 Three-dimensional display application with metasurface

3D display mainly includes naked-eye 3D display and near-eye 3D display, which is the development trend of future display equipment. The characteristics of 3D display devices, such as large field of view, large depth of field, high resolution, and miniaturization, are also basic factors affecting the optical performance of 3D displays, and traditional 3D display devices cannot have these characteristics simultaneously. Because metasurface has the advantages of excellent wavefront modulation ability and multi-function integration, it becomes an effective method to overcome the problems existing in traditional 3D display devices.

### 4.1 Three-dimensional holographic display

3D Holographic display is regarded as the most ideal 3D display technology. Since Gabor introduced the concept of holography in 1948 [153], a myriad of new holographic technologies have emerged. For example, pure-phase holograms and computer-generated hologram (CGH) are used to modulate the complex amplitude of light. However, in the above method, the process of each point or slice is independent, so it is difficult to present the occlusion and shadow information of the 3D image. To improve the quality of 3D display holograms, researchers have proposed some methods from the perspective of geometric optics, such as weighted intelligent pixels [154], [155], intermediate view reconstruction (IVR) [155], and Fourier spectrum-based novel look-up table (FS-NLUT) [156], which greatly improve the depth of field, angular spatial resolution and calculation speed of 3D holographic display. However, the above methods still cannot meet the requirements for multi-functioning and integrating optical components.

**Figure 10:**
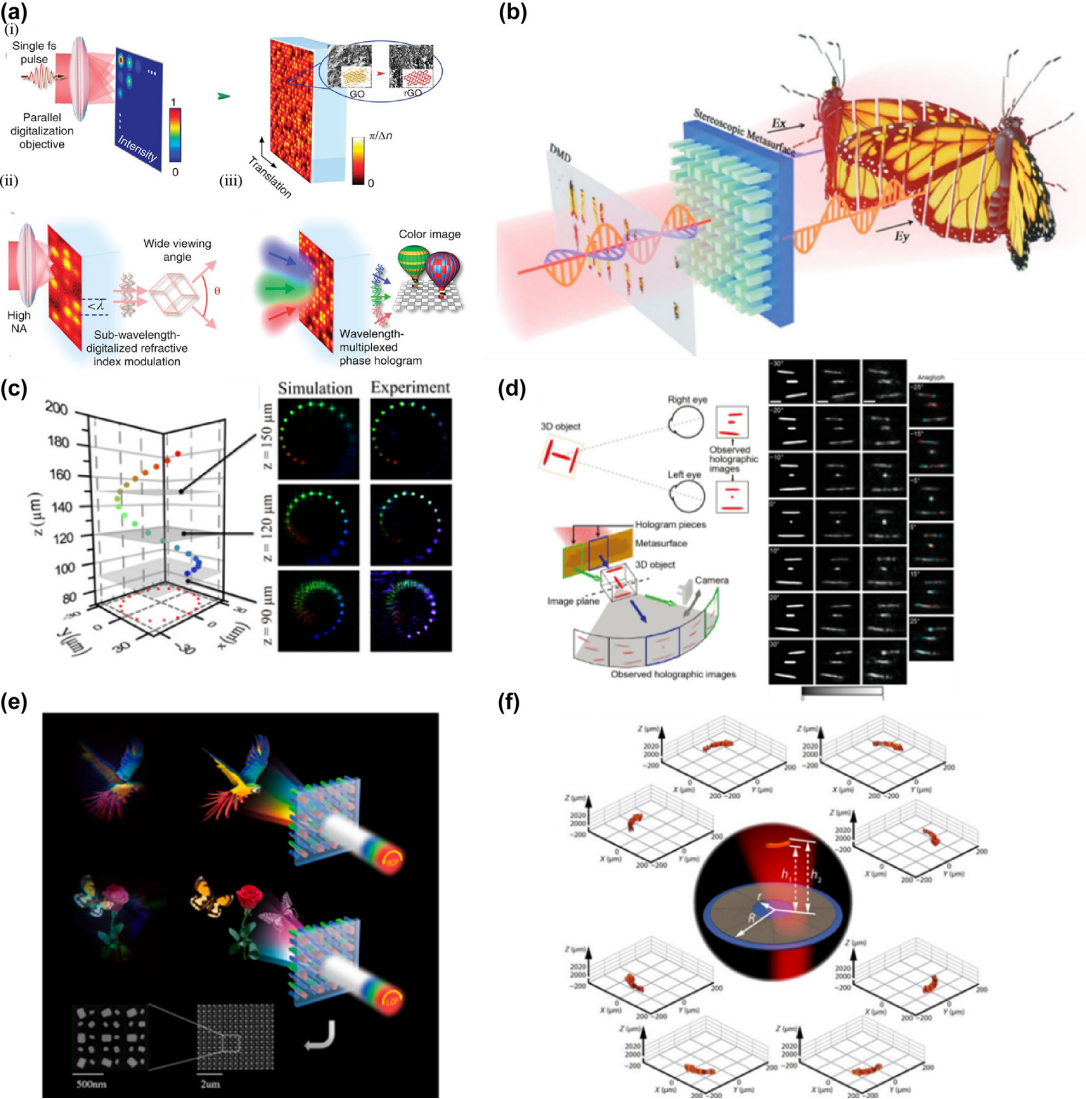
Advanced 3D Holographic Display and Meta-Hologram Techniques. a) (i) Schematic illustration of the optical digitalization of refractive-index/phase modulation by the thermal photoreduction using a single fs pulse, (ii) scheme of wide-angle 3D images by confining the photoreduction at a subwavelength scale through increasing the NA of the parallel digitalization objective and (iii) reconstruction of color objects through the wavelength-multiplexed phase hologram recorded in GO polymers. b) Illustration of the multifocal display system. c) Schematic diagram of the 3D object in the spatial coordinate and experimental results for the cross-sections at different *z* positions. d) Multi-view holographic stereogram. e) Schematic of the meta-hologram for spin-switched 3D full-color scenes. f) A dynamic 3D holographic display is achieved by a space channel selective meta-hologram. a) Reprinted from Ref. [158], under the terms of the Open Access Publishing Agreement; b) reprinted from Ref. [159], under the terms of the Open Access Publishing Agreement; c) reprinted from Ref. [160], under the terms of the Open Access Publishing Agreement; d) reprinted from Ref. [161], under the terms of the Open Access Publishing Agreement; e) reprinted from Ref. [162], with permission from ACS; f) reprinted from Ref. [163], under the terms of the Open Access Publishing Agreement.

Compared with traditional 3D holographic display components, metasurface design holograms have the advantages of high degrees of freedom, high spatial resolution, low crosstalk, and high bandwidth [157]. Li et al. created a large field of static view 3D hologram using a metasurface prepared by graphene oxide, A hologram that multiplexes wavelengths to realize a full-color image has also been achieved., and the phase modulation is programmed according to the holographic correlation of the 3D image [158]. As shown in Figure 10a, the combination of high numerical aperture (NA) objective and graphene oxide metasurface reduces the size of each focus to a sub-wavelength scale, thereby increasing the field of view angle. When the pixel size is reduced to 0.55 μm, the field of view angle is increased to 52°. The manufacturing method of graphene oxide polymers presents difficulties for large-scale preparation applications. Zhang et al. proposed a 3D multi-focus display method based on stereoscopic metasurface (SAS) [159]. Based on the Dammann optimization method and Fourier expansion with zone plate to generate phase profiles, the SMS can reassemble images from different planes to form 3D images with the correct depth cues (Figure 10b). Each layer of the 3D image is composed of different diffraction orders of SMS, and the energy of each diffraction order is uniform by the Dammann optimization method, which greatly reduces the crosstalk between each layer of planar images and improves the quality of 3D images. Low resolution and limited viewing angles compromise display quality, necessitating improvements to ensure optimal visual performance. When full-color holography is realized, the increase in operating wavelength range will cause crosstalk, affecting the hologram’s quality. Therefore, reducing the crosstalk between different colors in the wide band is key to realizing the color 3D hologram. Xiong et al. designed a geometric phase metasurface based on a wide-band slit antenna and applied the off-axis illumination method to metasurface holography [160]. Different wavelengths of light are slanted onto the metasurface at different design angles, and the output light is superimposed to form the color image, overcoming crosstalk between different wavelengths. As shown in Figure 10c, the metasurface reconstructs a color 3D spiral hologram in stereo space. Using a CCD camera to capture the holographic pattern at different *z*-axis positions, star maps of different colors can be obtained, verifying the depth information of the 3D hologram. This approach demands precise control over the angle at which light enters, a condition that may be challenging to meet accurately in upcoming display technologies.

The 3D holograms generated by the above metasurface are composed of several 2D holograms. The angle of the 3D images is fixed, and the 3D information cannot be observed in multiple viewing angles, which limits the application of these methods in the field of 3D display. Choi et al. proposed a metasurface stereo holographic technique that can provide binocular depth cues [161]. As shown in Figure 10d, the metasurface consists of several hologram blocks (green and blue boxes) that display 2D holographic projections of the corresponding directions of the target 3D image according to different viewing directions (green and blue arrows). A 3D image consisting of three rods with a volume of 25 μm × 25 μm × 25 μm is rendered; the three rods are located at different depths: the upper, middle, and lower rods are located at −12.5, 0, and + 12.5 μm, respectively, on the *z*-axis relative to the center of the target structure. The 3D hologram reconstructed by metasurface is composed of multiple hologram segments. 2D projection of the target 3D image is generated along the observation Angle parallel to the *X*-axis meridian, and perspective projection is rendered at 10° step length from the observation Angle of −30° to +30°, thus achieving the stereoscopic effect with binocular depth clue (Figure 10d). The 10-degree increment only allows for seven unique projection angles between −30° and 30°, which limits the amount of detail that can be displayed.

With the maturity of static holography, the research of dynamic 3D holography is particularly important. Huang et al. proposed a spin-switchable metasurface that can generate 3D full-color holograms [162]. Three different meta-atoms of the metasurface modulate three kinds of light: red, green, and blue, respectively. The phase distribution of the wavefront of the 3D hologram is retrieved using the GS algorithm, and the 3D full-color hologram is reconstructed by switching the spin of the circularly polarized light (Figure 10e). Significantly minimizes interference among colors of varying wavelengths. Creating images with three distinct focal depths has been accomplished, yet adding further images at varied depths demands substantial computational effort and correspondingly elevates the complexity of manufacturing. Gao et al. proposed a dynamic 3D metasurface holography with a large frame number and high frame rate based on spatial channel multiplexing, which can realize 228 different holographic frames and extremely high frame rate dynamic holographic display in the visible light range [163]. As shown in Figure 10f, a ring holographic superstructure surface has eight spatial channels, and each spatial channel reconstructs a 3D arrow in the three-dimensional space. A laser beam is used to illuminate each spatial channel, which can carry out a smooth dynamic 3D holographic display. This necessitates a laser light source characterized by high accuracy and rapid switching capabilities.

### 4.2 Three-dimensional light field display

Light field display is one of the most promising naked-eye 3D display technologies, which can be divided into geometric optics and micro-nano optics-based 3D displays. The light field display based on geometric optics generally uses a light field modulator composed of the refracting lens to project the light field information into space and form a 3D image of the light field with continuous parallax. However, the aberration of traditional lenses seriously affects the quality of reconstructed 3D images. To achieve the goal of high-quality 3D optical field display, researchers use adaptive periodic δ function array [164] and deep neural network [165] to sample the information. However, the problems of high manufacturing cost, high complexity, and large size of traditional display devices have not been solved.

**Figure 11:**
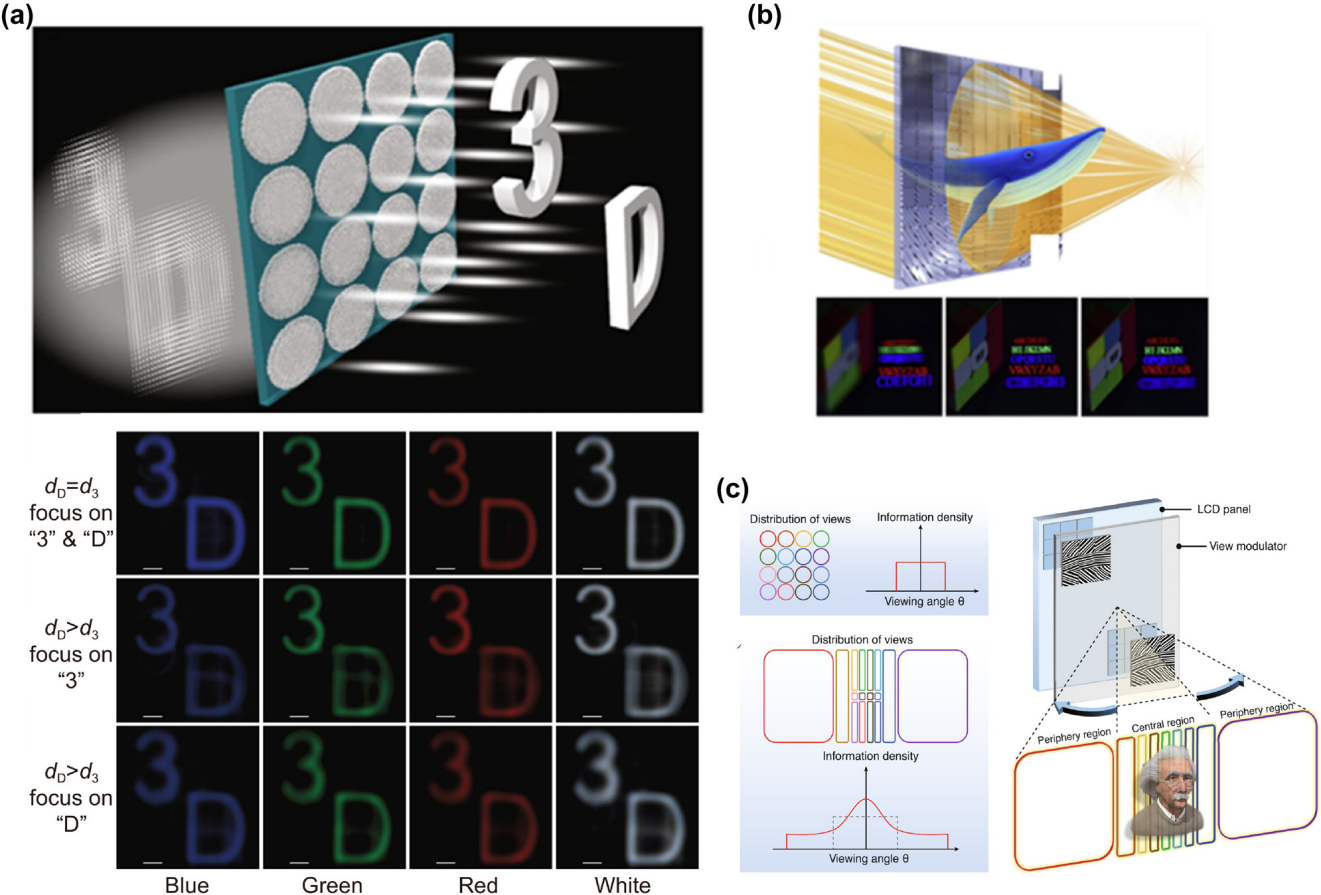
Advanced Light Field and Full-Color 3D Display Techniques Using Metasurfaces. a) Broadband achromatic matelens array for full-color light field display. b) Holographic sampling display based on metal gratings. (c) Spatial variable resolution light field 3D display. a) Reprinted from Ref. [166], under the terms of the Open Access Publishing Agreement; b) reprinted from Ref. [167], under the terms of the Open Access Publishing Agreement; c) reprinted from Ref. [168], under the terms of the Open Access Publishing Agreement.

From the micro and nano optics perspective, metasurface has a good aberration correction ability as a placal element, which is expected to make up for the shortcomings of traditional light field displays. Fan et al. designed a wide-band achromatic silicon nitride (SiN) metalens array capable of displaying full-color 3D scenes in a white light field [166]. As shown in Figure 11a, the 3D scene of “3” and “D” is encoded by the algorithm, and then the depth information of the image is reconstructed during the display of the light field. The metalens array consists of 60 × 60 polarization-insensitive metalenses with an effective achromatic refractive index ranging from 430 nm to 780 nm and a resolution close to the diffraction limit. In addition, the metalens array consists of only an ultra-thin 400 nm SiN layer, making it suitable for parallel processing of on-chip hybrid CMOS integration and optoelectronic information. However, the size of an achromatic metalens is often limited to a small scale because of the challenges in fabricating nanoposts with high aspect ratios and the constraints on the range of effective refractive indices. The naked-eye 3D display is the most ideal way to conform to the human eye’s viewing habits, but there are still many technical problems. In addition, the refresh rate and FOV of holographic 3D displays put forward high requirements on naked-eye 3D display devices. As a sampling form of holographic 3D, the light field 3D display can greatly reduce the amount of data and is the best display mode for naked-eye 3D display. Qiao et al. proposed a metasurface grating (MG) naked-eye holographic sampling 3D display [167]. The phase information of each MG pixel is used to form an aggregated viewpoint to improve the visual discomfort caused by crosstalk and boundary adjustment conflict. When the viewing points are closely aligned, the holographic sampling 3D display can be approximated as a continuous light field, the optical field 3D display. A large-scale ultra-structured surface grating field modulator is prepared by using two-dimensional grating and nanoimprint, and a 3D display screen is constructed compatible with the flat panel display. Dynamic full-color 3D display with a high refresh rate and no visual fatigue under LED white lighting, as shown in Figure 11b. However, the resolution of the existing display panel is limited, and the spatial resolution, angular resolution, and Angle of view of the 3D display of the light field will be mutually restricted. For example, to achieve a 3D display with 1 K spatial resolution and 3° vertical and horizontal angular resolution, a display panel with 50 K resolution is needed to achieve a 150° viewing angle, and existing display panels have difficulty meeting the resolution requirements.

Qiao et al. proposed and prepared an optical field 3D display with spatial variation resolution, which is composed of a hybrid arrangement of metasurface gratings with different shapes [168]. According to the information on the spatial variation of the observation frequency projection, the densely packed views are arranged in the center. In contrast, the sparsely arranged views are distributed in the periphery. The high-resolution image effect of smooth transition motion parallax can be presented in the central viewing angle area. At the same time, the sampling resolution is low in the peripheral viewing angle, but the viewing angle is enlarged, as shown in Figure 11c. This approach suppresses redundant depth information and expands FOV to a range comparable to 2D display panels by demonstrating full-color 3D display with video rates, with horizontal viewing angles up to 160°.

### 4.3 Three-dimensional near-eye display

AR and VR near-eye displays have received great attention from researchers as the latest next-generation 3D display technology. Wearable near-eye displays for AR and VR are also attracting much consumer interest. However, these near-eye displays only use light field intensity information and face performance challenges such as FOV, resolution, and depth of field range. In the pursuit of a better wearing experience, the challenges of near-eye displays often need to be balanced, and the display needs to be lightweight.

**Figure 12:**
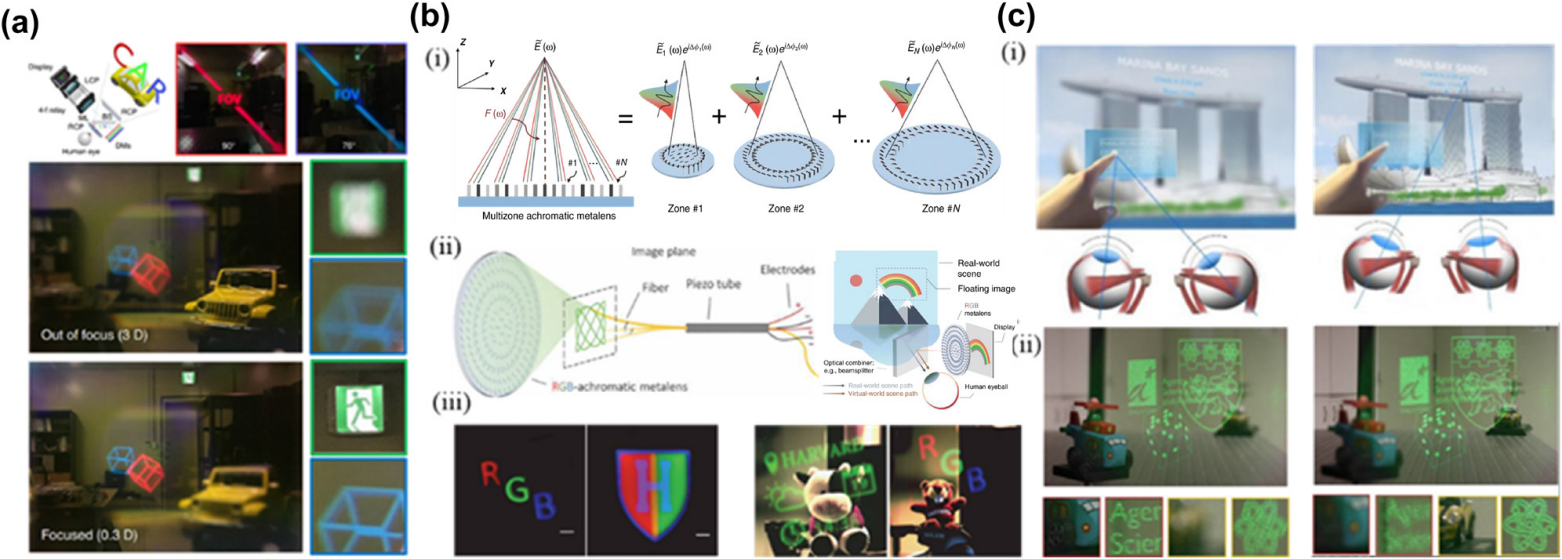
Metasurface-Based Near-Eye and Wide-FOV AR/VR Display Systems. a) Wide FOV eyepiece using metasurfaces in AR. b) (i) Schematic drawing of a multizone RGB-achromatic metalens showing achromatic focusing of RGB light coming from different lens locations, (ii) schematic illustration of the near-eye fiber scanning display and schematic illustration of the AR mod, and (iii) color VR imaging result. c) (i) Different locations of the scene have different focal depths, and (ii) it is focused at two diopters (0.5 m) and it is focused at 0.5 diopters (2 m). In this case, both real and virtual objects coexist in the scene. a) Reprinted from Ref. [166], under the terms of the Open Access Publishing Agreement; b) reprinted from Ref. [167], under the terms of the Open Access Publishing Agreement; c) reprinted from Ref. [168], under the terms of the Open Access Publishing Agreement.

**Figure 13:**
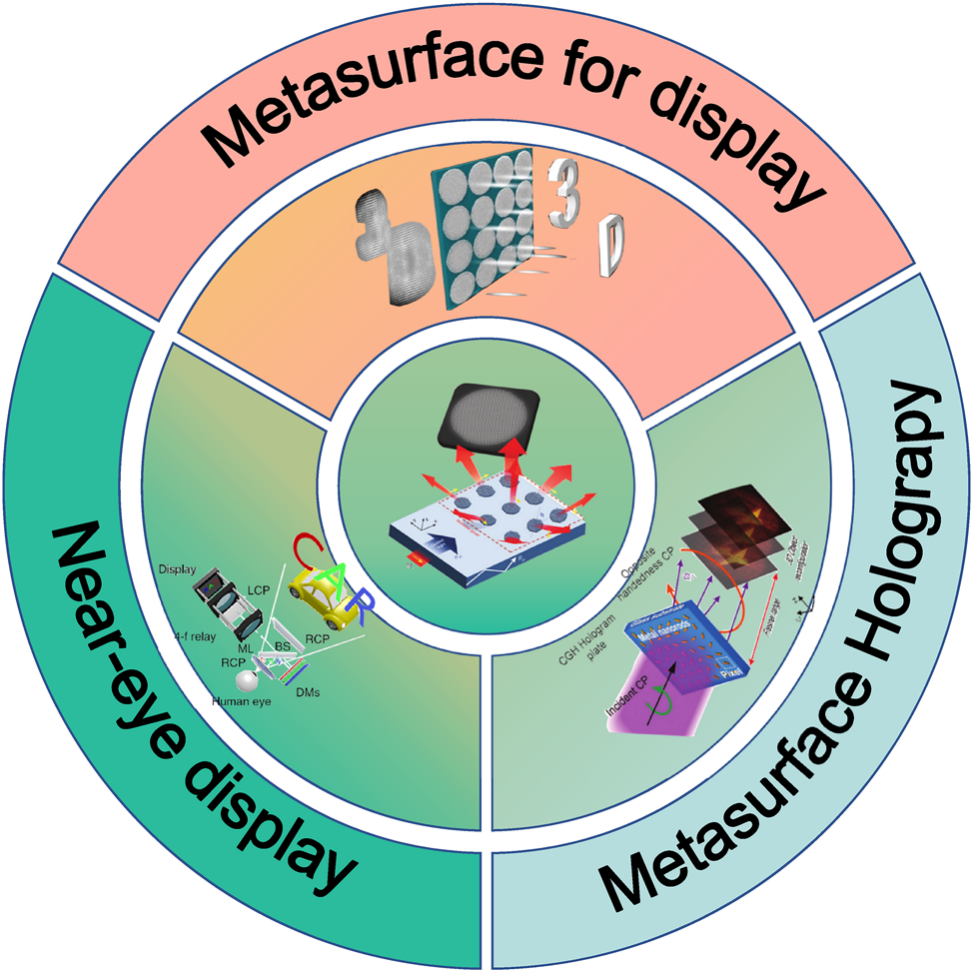
Summary of metasurface-enabled 3D display architectures, including computer-generated holography engines, light-field projection modules, and near-eye/retinal display stacks.

**Figure 14:**
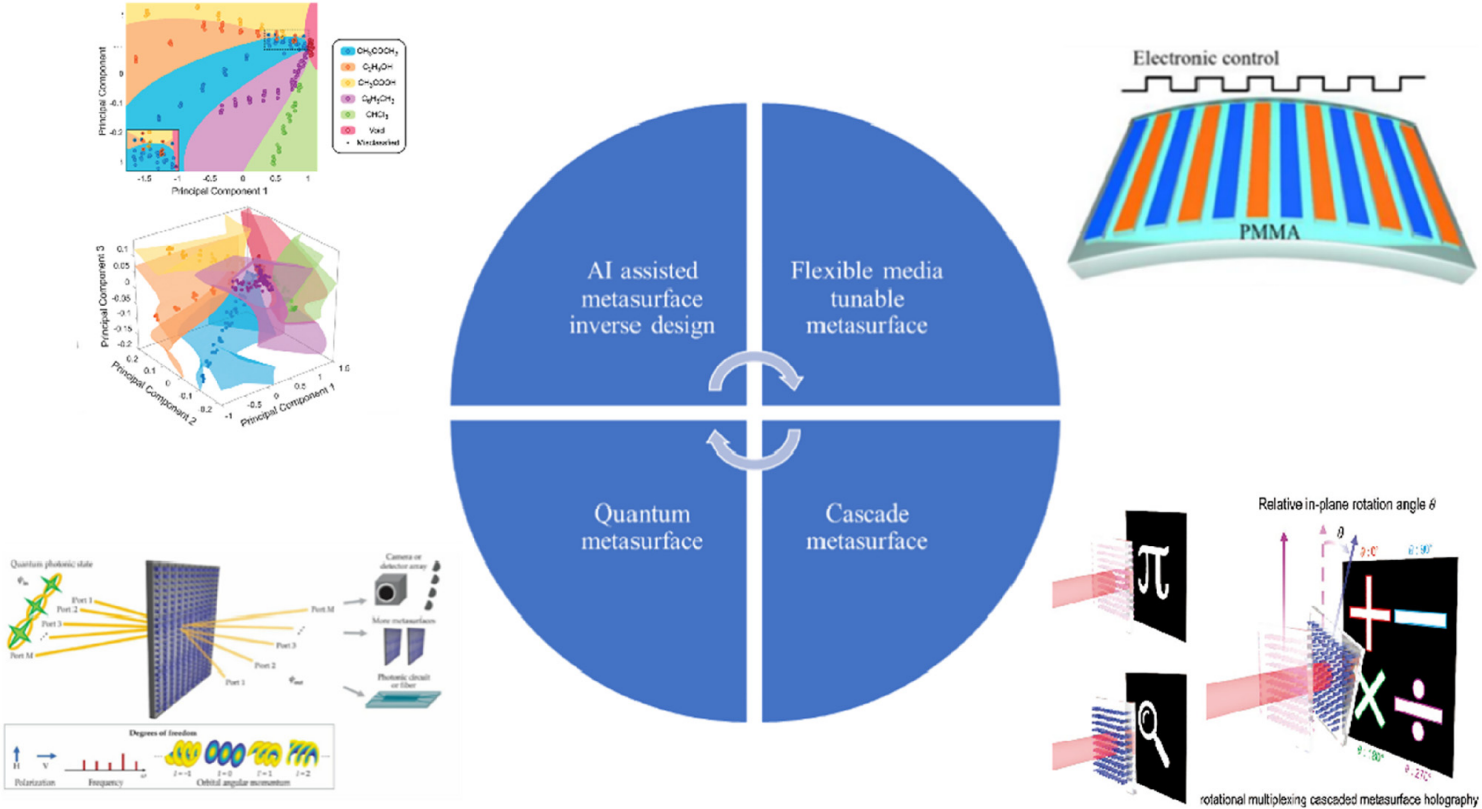
Technology readiness roadmap for metasurface-enabled 3D displays (2025–2030). Emerging research directions: AI-assisted inverse design, dynamically tunable multifunctional platforms, and quantum/cascaded metasurface architectures are mapped onto projected milestones in device efficiency, full-color operation, and wafer-level manufacturability. The top-left and bottom-left panels reprinted from Ref. [173] and Ref. [174], under the terms of the Open Access Publishing Agreement; the bottom-right panel is reprinted from Ref. [175], under the terms of the Open Access Publishing Agreement; all other parts were created by the authors.

The ability of arbitrary wavefront modulation and the ultra-thin feature of the metasurface has become an effective method to solve the problems of traditional near-eye displays. In 2018, Lee et al. proposed an AR near-eye display system based on high NA (NA 0.61) and large area (diameter 20 mm) transparent metalens [169]. As shown in Figure 12a, the display system consists of a beam projector, a 4f system, a beam splitter, a dichroic mirror, a circular polarizer, and a metalens eyepiece. The system makes the observation position of the human eye in front of the metalens, thereby increasing the FOV. The image is in the range of the focal length of the metalens, and the sharpness of each part of the image is adjusted by changing the position of the focal point. The color difference is corrected by changing the wavelength and imaging position of the dichroic mirror. The system realizes the high resolution and wide FOV of 90°of 3D full-color near-eye display. However, the above display method does not solve the color difference problem of metasurface in essence. With the metaverse concept, higher performance requirements are put forward for VR display devices, which require that the color difference problem existing on the metasurface be fundamentally solved. Li et al. proposed a multizone dispersion-engineered metalens to achieve the achromatic function of the metalens [170]. The schematic diagram of metalens is shown in Figure 12b; meta-atoms of the metalens can independently control the phase and dispersion of the light in the zone, so light in a single zone interferes at a focal spot for all design wavelengths. In addition, the inverse design method of moving asymptotically is used to optimize the regional transition position and phase discontinuity and finally obtain a large area of achromatic metalens. In contrast, the method of designing achromatic metalens by using a resonance mode of meta-atoms to compensate for dispersion can only achieve a metalens size of tens of microns. As proof, the researchers demonstrated a VR near-eye display system based on achromatic metalens with a diameter of 2 mm and fiber scanning (Figure 12b). The system creates grayscale images by applying multiple voltage levels to an acousto-optic modulator. Figure 12b shows an RGB letters three-color image and an H shield logo with seven colors taken in VR mode. These patterns have a pixel diameter of 1,300 nm, which is much smaller than the pixel size (∼8 μm) of micro-LCD on the market today. In addition, the achromatic metalens can be applied to AR devices (Figure 13b). In addition, the achromatic metalens can be applied to AR devices (Figure 13b), which allows virtual objects and real scenes to overlap. As shown in Figure 14b, the AR near-eye display composed of this metalens and fiber scanner mixes virtual images (gadget icons and three-color RGB letters) with real scenes. To enable near-eye displays to generate 3D scenes with higher resolution and continuous depth to improve user experience comfort, Song et al. proposed a compact holographic 3D near-eye display with a large exit pupil of 10 mm × 8.66 mm [171]. The AR near-eye display generates high-quality 3D scenes with ∼50 k active data points and a continuous depth of 0.5–2 m from large-pixel (>10^8^) Huygens’ metasurface holograms that overlap the real world (Figure 12c). As shown in Figure 12c, it has different display depths for different points in the virtual 3D scene, thus solving the convergence-adaptation conflict problem that exists in general display devices[172].

### 4.4 Scalable manufacturing for metasurface displays

Metasurface-based 3D displays require fabrication methods capable of bridging the gap between laboratory prototypes and commercial production. Traditional nanofabrication techniques, such as electron-beam lithography, offer high resolution but suffer from prohibitively low throughput and high cost for large-area devices. Indeed, the mass production of flat optical components like metalenses has been hindered by the high cost, low throughput, and limited patterning area of conventional methods [176], [177], [178], [179], [180], [181], [182]. Recent advances are addressing these challenges through the adoption of scalable nano-patterning techniques. These include roll-to-roll/roll-to-plate imprinting, nanoimprint lithography (NIL) utilizing advanced materials, and deep ultraviolet immersion lithography, enabling low-cost, large-area metasurface fabrication. Such approaches leverage established industrial processes to maintain sub-wavelength resolution while drastically improving throughput, positioning metasurface optics for increasing integration into real-world display engines.

Rho et al. introduced a roll-to-plate soft replication process for metasurface fabrication [179]. Utilizing an elastic mold, they achieved continuous imprinting on tape substrates followed by UV curing, thereby demonstrating chromatic aberration-compensated metalenses with a 1 cm aperture. The process achieved a production rate exceeding 10 m/min, extending optical disc manufacturing technology into the sub-100 nm regime. The high compliance of the soft mold significantly reduced detachment stress between the master mold and substrate, enabling direct replication onto plastic substrates.

Park et al. proposed a tape-assisted PER-NIL technique that eliminates residual layers by peeling off a tape post-nanoimprinting [177]. This approach solves the problem of adhesive residual layers caused by the etching rate disparity between the nanoparticles and resin in the medium. The method achieves a high refractive index (*n* ≈ 1.9) while reducing scattering noise by more than 60 % and narrowing the full width at half maximum (FWHM) of the color spectrum to 18 nm. Crucially, the entire process operates under ambient temperature and pressure, eliminating the need for vacuum deposition or etching equipment. This makes the technique particularly suitable for retrofitting existing display fabrication lines.

Kim et al. combined 193 nm ArF immersion lithography with wafer-scale UV-NIL to produce 12-inch masters featuring 40 nm resolution [181]. These masters were then used to batch-imprint 100 mm^2^ metalenses. By employing a conformal 5 nm ALD-TiO_2_ coating, the team dramatically increased device efficiency from 10 % to 90 %. This approach merges semiconductor-grade lithographic resolution with cost-effective NIL replication, positioning it as one of the most commercially viable pathways for AR/VR optical engines.

Table 1 compares key metrics of the three scalable fabrication pathways. Deep-UV lithography offers the finest resolution but at the highest cost; tape-assisted PER-NIL balances optical performance with low tooling cost; roll-based printing maximises area throughput for the lowest cost-per-area. Choosing or hybridising these platforms will determine which metasurface display form factors reach mass production first.

**Table 1:** Comparison of scalable fabrication techniques for metasurface displays.

Dimension	R2P/R2R	Tape-PER-NIL	DUV-IL+NIL
Throughput	Highest (continuous roll-to-roll)	Medium (limited by point-glue speed)	High (wafer batch processing)
Resolution	100 nm	80 nm (limited by PER particle size)	40 nm (ArF immersion)
Material system	Flexible polymers, Nylon	High-refractive index composite	TiO_2_/Al_2_O_3_, Si_3_N_4_
Post-processing compatibility	Roll-to-packaging	Requires low-temperature packaging	In-line with CMOS integration
Display compatibility	Large-area light-field screens, building facades	Structural color filters, micro-projection	VR/AR near-eye displays

Two critical hurdles in translating 3D display research to commercial products are device encapsulation and active electrical tunability. On the one hand, improper encapsulation of nanoscale optical components can lead to mechanical fragility and environmental degradation, yet adding protective layers often reduces optical efficiency by lowering refractive index contrastnature.com. Recent studies have tackled this by using high-refractive-index materials and spin-on-glass or polymer coatings to encapsulate metasurface optics without performance lossnature.com. This strategy yields robust, self-cleaning devices that withstand shock and contaminants, as demonstrated by encapsulated metalenses remaining undamaged after prolonged sonication with sandnature.com. On the other hand, achieving electrical tunability in 3D displays faces the trade-off between tuning speed and optical bandwidth. For example, liquid crystal layers offer convenient voltage control but respond on the millisecond scale and add bulk, whereas electro-optic or phase-change approaches can be faster but often with limited modulation depthscribd.com. To overcome this, researchers have integrated liquid crystals with metasurfaces to realize voltage-switchable beam splitters and metalenses operating across the visible spectrumpeeref.comlink.springer.com. Additionally, embedding Pockels-effect crystals like nano-patterned BaTiO_3_ has enabled solid-state focal length tuning with improved speedlink.springer.com. Solving these encapsulation and tunability challenges in tandem is key for future 3D display commercialization, and ongoing advances suggest that rugged packaging and active control can be co-developed without compromising optical performance.

Dynamic metasurfaces enable real-time optical modulation, with phase-change materials (PCMs) offering a transformative solution. GST225 (Ge_2_Sb_2_Te_5_)-based metasurfaces demonstrate ultrafast switching between plasmonic and dielectric states, achieving sub-millisecond response times for orbital angular momentum (OAM) vortex lasers [183]. This material’s low switching voltage and compatibility with CMOS processes make it ideal for energy-efficient displays. Recent studies have integrated GST225 with silicon photonic circuits to create tunable beam splitters and polarization controllers, highlighting its potential for compact augmented reality (AR) devices [184]. Compared to liquid crystal-integrated metasurfaces, PCM-based systems offer faster response times (less than 1 ms) [185].

Commercialization of metasurface displays requires addressing critical challenges in encapsulation and electrical tunability. High-index materials such as TiO_2_, commonly used in metasurface designs, are vulnerable to degradation under environmental stressors like humidity and temperature fluctuations. To mitigate this, anti-moisture encapsulation strategies such as parylene coatings or multilayer Al_2_O_3_/SiO_2_ films have been developed, demonstrating operational lifetimes exceeding 10 years in accelerated aging tests [186]. However, scaling these techniques to large-area, cost-effective production remains challenging due to throughput limitations and fabrication complexity [187]. Electrical tunability is another key hurdle for dynamic applications. While phase-change materials (PCMs) like GST225 enable ultrafast optical switching, their integration with drive electronics introduces technical complexity. Liquid crystal-integrated metasurfaces offer an alternative but suffer from slower response times (∼10 ms), limiting their suitability for real-time applications [188]. Hybrid approaches combining electro-optic polymers with PCM layers are being explored to balance speed, tunability, and scalability [189]. **Comparatively, metasurface displays outperform conventional LCDs** **in thickness reduction and response time** but LCDs benefit from mature, high-throughput manufacturing infrastructure. In contrast, metasurface-based tunability relies on niche fabrication processes, creating a significant barrier to mass production [187]. Addressing these challenges through advanced encapsulation, hybrid tuning mechanisms, and scalable manufacturing is essential for translating metasurface technologies into practical display systems.

Cost analyses indicate that step-and-repeat nano-imprint lithography combined with ArF immersion can reduce optics fabrication costs to below $0.03 cm^−2^ when scaled to 300 mm wafers [190]. Pilot production runs integrating metasurface backplanes with µLEDs have already yielded 1.5-inch full-color display engines operating at 120 Hz [191], while roll-to-roll PER-NIL has achieved metre-scale foils with costs under $5 m^−2^ [192].

## 5 Conclusion and outlook

In this paper, we focus on how metasurface generates high-quality holograms and structural colors, as well as its applications in the field of 3D display. First, we introduce the basic principle of metasurface wavefront modulation. Then, we introduce in detail the multiplex method of metasurface generating holograms and the method of generating high-quality structure color, which makes metasurface application in the 3D display field possible. As research hotspot in the field of 3D display, we introduce the application of metasurface in holographic display, light field display, and near-eye display in detail and explain the methods to solve the problems of traditional 3D display devices from the perspective of micro-nano optics. With the metasurface’s powerful light field modulation capability and ultra-miniaturization, the new metasurface 3D display system has better imaging quality, larger FOV, and lighter weight. However, to achieve the ultimate commercialization of metasurfaces in the display field, some challenges remain to overcome.

To smoothly display optical information, it is essentially necessary to require the number of frame rates to approach infinity. In this regard, some progress has been made through OAM multiplexed metasurface holography [61], but the holographic displays in science fiction movies are still a long way off. Because of the integrated characteristics of the metasurface, its manufacturing difficulty and manufacturing cost increase. In terms of material selection for meta-holography and color printing methods, TiO_2_ with large metal loss and low loss can greatly improve work efficiency. However, TiO_2_ preparation by atomic layer deposition technology requires a lot of time, and the diffraction efficiency of silicon metasurface is low, which goes against the purpose of commercialization. Therefore, more efficient micro-nano processing methods and better materials are needed to solve the production problems. In the field of 3D display, the numerical aperture, efficiency, and working bandwidth of achromatic metalens will be mutually restricted, and the 3D holographic display realized by metasurface is only the projection of 2D hologram with depth information, rather than continuous 3D holography [193]. With the increase in design complexity of the metasurface, crosstalk between different channels and work efficiency need to be solved. Therefore, the metasurface still needs continuous improvement and breakthroughs in terms of performance improvement. Currently, the methods to solve the problems of narrow operating frequency and angle dependence of metasurface often need to combine metasurface with traditional optical components [158], [193]. However, this integration method often has high requirements, such as the size of the metasurface being large enough, difficulty in manufacturing, and high production costs. Therefore, it is necessary to consider optimizing the properties of the metasurface itself to solve the existing problems. To improve the performance of metasurface under tshe premise of existing technology and reduce its design complexity, the future development direction of metasurface includes artificial intelligence (AI) assisted metasurface inverse design, adjoint algorithm-based optimization, flexible media tunable metasurface, cascade metasurface and quantum metasurface. In recent years, the inverse approach of AI-assisted design of metasurfaces, capable of designing arbitrary meta-atomic structures with higher DOFs, has been validated by advanced simulation optimization [194], [195] machine learning [196], and deep learning [197], [198]. AI-driven metasurface design has advanced rapidly through the integration of deep learning techniques such as generative adversarial networks (GANs) and reinforcement learning (RL) [199], [200], [201], [202], [203], [204], [205]. GANs have been employed to generate highly complex, non-intuitive nanostructures for tasks like holography and beam shaping, achieving superior performance beyond traditional inverse design methods. For example, Jiang et al. demonstrated that GAN-generated diffractive metasurfaces could produce custom holographic projections with enhanced accuracy and efficiency [204]. In parallel, RL algorithms enable dynamic optimization of metasurface performance – such as polarization control or light efficiency – via iterative feedback, as shown by Sun et al., who optimized structural parameters while reducing fabrication complexity [205]. Together, these AI frameworks provide powerful, scalable solutions for tailoring metasurface functionalities in next-generation display and optical systems. At present, various optical systems based on metasurface designs have been studied, such as light field cameras [166], [206], polarization cameras [207], [208], and spectrometers [209]. However, the function of the static single-layer metasurface applied to the optical system is fixed, which limits the flexibility and versatility of optical systems. The tunable multifunctional integration of metasurface is the key to broadening its application range [210]. The use of flexible or adjustable dielectric materials to design metasurfaces of various types of geometry enables more novel new perspectives for metasurface design [69], [145], [211]. The design DOF of a single-layer metasurface is still inherently limited, and the information cannot be physically split once the design is complete. In recent years, cascading metasurfaces that can replace, translate, or rotate their own components have been rapidly developed [212], [213], [214], [215]. It can flexibly combine and split the encoded information and, combined with other novel technologies, greatly accelerate the commercialization process of the metasurface. Quantum metasurfaces planar arrays that coherently shape single photons or entangled photon pairs offer a route to ultralow-power, shot-noise-limited display engines. Recent demonstrations of entangled-photon holography and single-photon metalens arrays indicate that harnessing coherence and entanglement can enable noise-free holographic projection, intrinsic data encryption, and sub-attojoule pixel switching. Although still at proof-of-concept stage, these results map directly onto the efficiency, field-of-view and color-depth benchmarks outlined in this review, and thus merit concise inclusion in our commercialization roadmap [193], [216], [217]. With the development of nanomanufacturing technology and the emergence of new design methods, efforts will be made to make metasurface-based display technology the next generation of display technology.
